# Two-stage posture enhancement and stability modelling for pre-fall risk detection in athletic movements

**DOI:** 10.3389/fbioe.2026.1867362

**Published:** 2026-08-07

**Authors:** Lei He, Shiping Wang, Hae-in Seo, Qi Shen, Yuang Cao, Mengqiang Hu, Shihong Zhang, Huiteng Xu, Jianfei Wen, Junjun Gun

**Affiliations:** 1 Department of Physical Education, Woosuk University, Wanju-gun, Jeollabuk-do, Republic of Korea; 2 Department of Recreation and Leisure Sports, College of Sports Science, Dankook University, Yongin-si, Republic of Korea; 3 College of Arts & Physical Education, Gangwon University, Gangneung-si, Republic of Korea; 4 School of Physical Education, Hefei Normal University, Economic and Technological Development Zone, Hefei, Anhui, China

**Keywords:** fall risk, posture assessment, prediction model, sports injury, two-stage framework

## Abstract

**Background:**

In sports training and athletic performance, fall risk degrades performance and elevates injury incidence, making early warning of pre-fall states critical. Monocular 3D pose estimation often yields incomplete or biomechanically inconsistent skeletons due to occlusion, blur, and detector noise, limiting reliable monitoring of movement stability and quality.

**Method:**

We propose a two-stage deep learning framework: 1. RePoseNet: Refines initially estimated 3D skeleton sequences generated by a pretrained monocular pose estimator, rather than performing monocular 3D pose estimation from scratch. It repairs missing or unreliable joints, suppresses temporal jitter, and enforces biomechanical plausibility through joint symmetry, limb-length consistency, and joint-angle constraints. 2. StaFallNet: Integrates stability metrics—Step Frequency Variation Rate (SFVR), Trunk Leaning Angle (TLA), and Stride Variability (SV)—into a dynamic graph network to model spatial coordination and temporal dynamics for frame-level and event-level pre-fall risk prediction.

**Methodology Details:**

RePoseNet combines GRU-based temporal repair, cross-joint attention, and biomechanical refinement (limb symmetry, joint-angle limits). StaFallNet extracts biomechanical features (SFVR, TLA, SV), constructs dynamic joint graphs modulated by stability cues, and uses attention pooling for risk classification. Evaluation: Tested on a fall-related subset of UCF101 with manually annotated frame-level labels. Pre-fall frames were defined as frames within a fixed temporal window before the visually identified fall onset, while non-risk frames were sampled from stable movement periods.

**Results:**

RePoseNet improves temporal smoothness and biomechanical plausibility under controlled skeleton-level corruption. Reference-based Mean Per Joint Position Error and PCK evaluations indicate that the refined skeletons remain consistent with the initial pseudo-3D pose estimates while reducing temporal instability; these metrics are not interpreted as evidence of improved absolute 3D pose accuracy. More importantly, downstream experiments demonstrate that the refined skeletons provide more reliable inputs for stability-aware fall-risk prediction.

**Conclusion:**

This work offers a robust, interpretable approach for fall-risk prediction in sports. It provides actionable insights for performance evaluation, training intervention, and injury prevention, bridging computational modeling with biomechanical principles.

## Introduction

In competitive sport environments, athletes regularly experience sudden perturbations during rapid changes of direction, deceleration, or physical contact, and these perturbations frequently push the limits of postural control. Most prior work on fall risk or balance instability focuses on older adults or clinical populations rather than addressing the high-velocity multi-joint dynamics characteristic of sport. A recent experimental study showed that when healthy young adults standing on a treadmill are subjected to perturbations of increasing peak velocity, the proportion of falls increases markedly (Shin et al., 2024). Another investigation demonstrated that backward slip perturbations induced by a split-belt treadmill with greater speed yield significantly higher fall rates and loading forces in young adults (Lee et al., 2025). Such findings imply that small changes in external load intensity can destabilize systems that otherwise appear stable (Shokouhi et al., 2024; Wang et al., 2024b). In the domain of athletic movement, where perturbations are more frequent larger and more complex, maintaining robust real-time stability is thus not trivial. It follows that developing methods capable of monitoring and interpreting instability in sports motion holds critical scientific and practical value.

In this study, the term “fall risk” specifically refers to instability patterns arising primarily from intrinsic movement-control deficits during athletic motion, rather than falls caused by unpredictable external perturbations such as direct collision, environmental slipping, or opponent contact. Typical examples include loss of balance during landing, unstable deceleration, excessive trunk sway, asymmetric lower-limb coordination, and failed postural recovery after rapid directional transitions. Accordingly, the proposed framework focuses on detecting biomechanical instability signatures that emerge within the athlete’s own movement dynamics. The extracted kinematic features—including step-frequency fluctuation, trunk leaning behavior, and stride variability—are therefore designed to characterize deterioration in postural coordination and locomotor stability preceding these internally driven fall events. Importantly, the proposed framework is not restricted to conventional gait activities. For non-gait athletic actions such as diving, trampoline jumping, pole vaulting, and skateboarding, instability is defined as a progressive loss of whole-body postural control, impaired landing preparation, abnormal trunk orientation, or unsuccessful balance recovery during movement execution. Therefore, the extracted stability features are interpreted as generic movement-stability indicators rather than gait-exclusive descriptors.

Many recent studies have explored how deep learning and perturbation-based methods aid in balance and movement stability analysis. Chen et al. developed a wearable system combining sensors and neural networks to estimate vertical ground reaction forces during movements after anterior cruciate ligament reconstruction and demonstrated its promise in detecting subtle biomechanical deviations (Chen et al., 2025). The efficacy of perturbation-based balance training has also attracted attention, with a systematic review showing that such training yields significant improvements in reactive balance control and gait recovery among older adults (Schoeneberg et al., 2024). In addition, hybrid deep learning models incorporating alignment and subject-specific features have achieved enhanced accuracy in predicting biomechanical variables such as ground reaction force during walking (Sugiarto et al., 2025). These works reflect a trend toward combining sensor data, biomechanical modeling, and data-driven frameworks in movement science. Yet almost all of them remain within contexts of moderate speed or clinical/rehabilitation populations. Their direct applicability to athletic motion with high velocities, abrupt transitions, and complex perturbations is still limited. Recent advances in biomechanical computing have further highlighted a growing trend toward mechanism-aware deep learning frameworks that integrate data-driven modeling with explicit biomechanical principles. For example, recent studies on ligament mechanics have combined deep learning with computational biomechanical models to predict ligament fatigue failure risk and loading mechanisms under complex motion conditions (Xu et al., 2025; Xu et al., 2026). These approaches demonstrate that purely data-driven learning may be insufficient for modeling physiologically meaningful movement behaviors, and that integrating biomechanical constraints or mechanistic priors can substantially improve interpretability, robustness, and physical consistency. This emerging direction motivates the present work, where biomechanical plausibility constraints and stability-aware graph adaptation are explicitly incorporated into skeleton refinement and fall-risk prediction for athletic motion analysis.

Despite these promising advances, significant gaps still impede robust pre-fall detection in athletic motion. First, many existing pose estimation methods and datasets are not designed for high-acceleration, complex sports actions. For example, the recently proposed AthletePose3D dataset shows that models trained on standard datasets yield very large errors, and only after fine-tuning on sports motions can the error be reduced to 65 mm (Yeung et al., 2025). Second, few reconstruction methods explicitly embed biomechanical priors or enforce temporal consistency, leaving the recovered skeletons vulnerable to unrealistic joint displacements or jitter under perturbations. Some works in motion biomechanics have used optimization constraints to simulate postural transitions under perturbation (Hong and Park, 2025), but they rarely integrate such constraints into deep learning pipelines for pose reconstruction. Third, instability or fall-risk prediction models often treat each joint or trajectory independently and do not model dynamically evolving inter-joint coordination, especially under transient perturbations. A recent review in sports biomechanics notes that explainability and modeling of inter-joint dependencies remain central challenges in applying machine learning to fast movements (Dindorf et al., 2024). These limitations suggest that achieving reliable pre-fall prediction in athletic motion will require methods that jointly address pose fidelity, biomechanical realism, and coordination modeling under dynamics.

To address these challenges, we propose a two-stage deep learning framework that integrates skeleton refinement with stability-aware prediction for athletic motion. In the first stage, RePoseNet is designed as a post-estimation refinement module. Specifically, RGB videos are first processed by an existing monocular pose estimation pipeline to obtain initial 3D skeleton sequences. RePoseNet then refines these initially estimated skeletons by repairing missing or unreliable joints, reducing temporal jitter, and enforcing biomechanical plausibility through constraints on joint symmetry, limb length, and joint angle ranges. Therefore, RePoseNet is not intended to replace monocular 3D pose estimation models; rather, it improves the reliability and anatomical consistency of their outputs for downstream stability analysis.

The second stage constructs a dynamic graph based on the refined skeletons and incorporates stability features such as trunk posture, step frequency, and stride variability. By jointly modeling spatial coordination and temporal dynamics, the framework generates frame-level instability estimation and event-level early warning and highlights the role of stability-critical joints. The novelty of this work does not lie in introducing entirely new backbone architectures such as GRUs, attention mechanisms, or graph convolutions individually, since these techniques are already widely studied. Instead, the main innovation lies in how these components are reformulated and jointly integrated for biomechanically grounded pre-fall risk analysis in athletic motion. First, RePoseNet introduces a post-estimation refinement paradigm specifically designed for sports-motion skeleton sequences, where temporal repair, cross-joint dependency modeling, and biomechanical constraints are unified into a single optimization framework. Unlike conventional pose smoothing or generic temporal filtering approaches, RePoseNet explicitly targets anatomically implausible motion artifacts caused by rapid athletic movement, occlusion, and unstable body transitions. Second, StaFallNet proposes a stability-aware dynamic graph modeling strategy in which biomechanical instability indicators directly modulate graph topology construction. Rather than using a fixed anatomical graph, the proposed method adaptively adjusts inter-joint connectivity according to motion stability cues such as trunk leaning behavior, stride variability, and gait-frequency fluctuation, enabling the network to capture transient coordination breakdown patterns preceding falls. Finally, the proposed framework establishes a closed-loop pipeline linking skeleton refinement and stability-aware prediction, allowing biomechanical plausibility enhancement and risk inference to operate in a mutually consistent manner for athletic-motion analysis. More importantly, the proposed framework explicitly bridges biomechanical priors and data-driven representation learning through a constraint-guided optimization strategy. In RePoseNet, biomechanical constraints are not treated as post hoc corrections, but are incorporated into the learning objective as structural regularization terms that guide temporal feature reconstruction toward anatomically plausible motion patterns. Similarly, in StaFallNet, biomechanical stability indicators are not merely appended as auxiliary handcrafted features; instead, they directly modulate graph topology adaptation and joint-level attention weighting, thereby influencing how spatial-temporal dependencies are learned under unstable movement conditions. This design enables biomechanical knowledge to function as an inductive bias for representation learning, improving physical consistency, interpretability, and robustness under noisy athletic-motion scenarios.

### Related work

In recent years, there has been rapid growth in methods combining computer vision, biomechanics, and learning to analyze human motion stability and risk. While pose estimation and motion modeling techniques have matured, their use in predicting instability in high-speed athletic motion is still emerging. Traditional pose systems struggle with blur, occlusion, and rapid dynamics, especially in sports scenarios. Accordingly, researchers have begun to address these gaps by creating new datasets, refining pose models with anatomical constraints, and applying graph/temporal models for fall or instability prediction.

Much researches focus on creating datasets and enhancing pose estimation tailored to sports. The AthletePose3D dataset was published in 2025 to specifically capture high-acceleration athletic movements. Models trained on conventional datasets produce errors on it, whereas fine-tuning reduces error to 65 mm, confirming a large domain gap in sports motion (Yeung et al., 2025). Transformer-based sports pose methods have also emerged. KASportsFormer integrates kinematic anatomy priors into a transformer model for short sports scenes and shows robustness to occlusion and blur, reaching error levels like 58 mm on SportsPose and 34.3 mm on WorldPose (Yin et al., 2025). In combat or multi-person sports, physics-based optimization and motion smoothing are integrated into pose pipelines, such as in Multi-person Physics-based Pose Estimation for Combat Sports, which blends triangulation, smoothing, and kinematic regularization to better handle occlusion and interactions (Khoiee et al., 2025). Beyond pose, many methods aim to predict fall events or instability using graph or spatio-temporal neural networks. Wang et al. proposed a spatio-temporal graph neural network that encodes latent relations among joints to predict falls from pose sequences (Wang et al., 2024a). Liu et al. developed a real-time system using multiple motion sensors and a spatio-temporal graph model to anticipate falls (Liu et al., 2025). The hybrid model Anticipatory Fall Detection decouples motion prediction and gait classification using DGNN + LSTM to detect transient states preceding a fall (Cho et al., 2025). In the domain of biomechanics and postural control, studies have applied deep networks to posture signals. For instance, Robles Cruz et al. explored trunk movement patterns using deep learning to classify fall risk in balance tasks (Robles Cruz et al., 2024). In action segmentation or motion temporal modeling, 3D pose–based temporal segmentation methods have been developed, which help handle fine-grained motion dynamics (Tanaka et al., 2024). HRPose demonstrates that combining high-resolution feature backbones with SinglePose modules can significantly improve keypoint accuracy in athletic motion contexts, achieving lower error under dynamic conditions (Han et al., 2025). In the realm of sports-aware pose estimation, Xi et al. propose enhancements suited for athletic environments, showing meaningful gains in motion tracking reliability in training settings (Xi et al., 2024). Meanwhile, the validation work by Fukushima et al. comparing pose estimation against marker-based systems reveals nonnegligible joint-angle errors—on average 9.7°—under real athletic movements, indicating that biomechanically consistent reconstruction is still needed (Fukushima et al., 2024). On the injury prediction side, reviews such as that by Leckey et al. survey current machine learning approaches to sport injury risk and observe gaps in model interpretability and context awareness (Leckey et al., 2025). Similarly, predictive frameworks in sports deep learning reveal that integrated pipelines combining pose, stability features, and temporal modeling are seldom explored (Sadr et al., 2025).

In summary, existing research has established important foundations through the development of sports-specific datasets, anatomically informed pose estimation methods, and graph-based instability prediction frameworks. However, most approaches address these aspects in isolation, which limits their applicability to the fast and perturbed motions that characterize athletic performance.

## Materials and methods

### Overall pipeline

For clarity, t denotes the temporal frame index, j denotes the joint index, and all 3D joint coordinates are represented in Cartesian space as $\left(x,y,z\right)$ . To avoid ambiguity regarding the role of each component, we clarify the input–output structure of the proposed framework. The complete pipeline consists of three sequential steps. First, RGB video frames are processed by a pretrained monocular pose estimation pipeline, where RTMPose-L detects 2D keypoints and VideoPose3D lifts them into pseudo-3D skeleton sequences in the COCO-17 joint format. Second, RePoseNet operates on these initially estimated pseudo-3D skeletons as a post-estimation refinement module. Its objective is to repair missing or unreliable joints, improve temporal consistency, and impose biomechanical constraints; it does not estimate 3D pose directly from RGB frames. Third, the refined skeleton sequence is passed into StaFallNet, which extracts stability-related features and performs frame-level pre-fall risk classification.

Because the pseudo-3D skeletons follow the COCO-17 joint format, several biomechanically meaningful virtual joints are additionally derived for stability analysis.

Specifically,

$$x_{\text{pelvis}}=\frac{x_{{\text{hip}}_{L}}+x_{{\text{hip}}_{R}}}{2}$$

is defined as the midpoint of the left and right hip joints.

$$x_{\text{neck}}=\frac{x_{{\text{shoulder}}_{L}}+x_{{\text{shoulder}}_{R}}}{2}$$

is defined as the midpoint of the left and right shoulder joints.

$$x_{\text{chest}}=\frac{x_{\text{neck}}+x_{\text{pelvis}}}{2}$$

is defined as the midpoint between the neck and pelvis.

$$x_{\text{spine}}=\frac{x_{\text{chest}}+x_{\text{pelvis}}}{2}$$

is used as an approximate trunk-center representation.

These virtual joints are not part of the original COCO-17 keypoint set and are generated only for biomechanical feature computation, graph construction, and interpretability analysis.

Formally, the input to RePoseNet is denoted as $X\in R^{T\times J\times 3}$ , where T is the number of frames and J is the number of anatomical joints. Because UCF101 does not provide motion-capture-quality 3D joint annotations, the initial 3D skeletons generated by the pretrained monocular pose estimation pipeline are also used as pseudo-3D reference poses for the reference-based RePoseNet evaluation. Consequently, the reported MPJPE and PCK values should not be interpreted as measures of absolute pose accuracy improvement. Instead, they quantify reconstruction consistency with the pseudo-reference skeletons under controlled corruption and refinement settings. The primary purpose of RePoseNet is to improve temporal stability and biomechanical plausibility for downstream fall-risk modeling. The output is a refined skeleton sequence $\hat{X}\in R^{T\times J\times 3}$ , which is used by StaFallNet for stability-aware dynamic graph modeling.

### Datasets

To evaluate the effectiveness of RePoseNet and StaFallNet, we constructed a fall-related subset from UCF101 (Soomro et al., 2012). UCF101 is an action recognition dataset containing 13,320 videos from 101 action categories. We acknowledge that UCF101 was originally designed for general action recognition rather than clinical fall-risk prediction or medical-grade balance assessment. Therefore, the purpose of using UCF101 in this study is not to claim direct clinical diagnostic validity. Instead, it serves as an exploratory sports-motion benchmark for evaluating whether pose refinement and stability-aware graph modeling can detect visible pre-fall instability patterns in challenging unconstrained videos. The dataset contains diverse camera viewpoints, background variations, motion blur, and fast athletic movements, making it useful for testing the robustness of skeleton-based modeling under realistic visual noise. However, it lacks standardized clinical fall annotations, controlled biomechanical measurements, motion-capture reference trajectories, and subject-level clinical information. These limitations are explicitly considered when interpreting the results. Since UCF101 does not provide an official fall-risk subset, we manually screened sport- and motion-related categories and selected videos in which visible loss of balance, failed landing, body descent, or body-ground contact occurred.

The selected subset included videos from the following action categories: Basketball, BasketballDunk, Diving, HighJump, LongJump, PoleVault, SkateBoarding, Skiing, SoccerJuggling, TrampolineJumping, and VolleyballSpiking. These categories were selected because they contain rapid direction changes, jumping or landing, aerial motion, slipping, or collision-like movement patterns. Most selected scenarios involve instability generated during self-initiated athletic actions such as landing, turning, jumping, or rapid deceleration, making them appropriate for studying intrinsic movement-related fall risk rather than externally forced falls caused by direct collisions or environmental hazards. Videos without visible instability or fall-related motion were used only as non-risk reference sequences when stable movement periods were required. The final subset contained 214 videos and 214 annotated fall events, with video durations ranging from 2.1 to 7.8 s.

For each video, 2D keypoints are first extracted using the pretrained RTMPose-L detector and subsequently lifted into pseudo-3D skeleton sequences using VideoPose3D. The resulting skeletons follow the COCO-17 joint format and are represented as frame-wise 3D joint coordinates. Since UCF101 is an action recognition dataset and does not provide true 3D joint annotations, these estimated 3D skeletons are treated as pseudo-3D reference poses rather than motion-capture-quality ground truth.

These pseudo-3D skeletons serve two purposes. First, they provide the initial input to RePoseNet, which refines the skeleton trajectories by recovering missing joints, reducing temporal discontinuities, and correcting biomechanically implausible configurations. Second, they serve as reference skeletons for evaluating RePoseNet using MPJPE and PCK. Therefore, the pose-related evaluation in this study reflects refinement consistency relative to the pretrained estimator output, rather than absolute 3D pose reconstruction accuracy.

The refined skeleton trajectories are then used to compute three biomechanically meaningful stability features—Step Frequency Variation Rate (SFVR), Trunk Leaning Angle (TLA), and Stride Variability (SV)—which serve as auxiliary inputs to the stability-aware graph-based prediction module.

### Pretrained pose estimation pipeline

Since UCF101 does not provide ground-truth 3D skeleton annotations, an existing monocular pose estimation pipeline was used to generate pseudo-3D skeleton sequences.

Specifically, RTMPose-L was first employed to detect 2D human keypoints from RGB frames. The detected 2D skeletons were represented using the COCO-17 joint format.

Subsequently, VideoPose3D was used to lift the 2D keypoints into pseudo-3D joint coordinates through temporal convolutional modeling.

The generated pseudo-3D skeleton sequences served as:

1. the initial input to RePoseNet;
2. the pseudo-reference skeletons used in the reference-based MPJPE and PCK evaluation protocol.

No additional fine-tuning of RTMPose-L or VideoPose3D was performed on the UCF101 subset. Both models were used with publicly available pretrained weights.

### Frame-level pre-fall label construction

Since UCF101 does not provide native frame-level pre-fall annotations, we manually constructed binary labels for StaFallNet. For each selected fall-related video, fall onset was defined according to the action context. For locomotion-related actions (e.g., Basketball, BasketballDunk, Skiing, and SkateBoarding), fall onset was defined as the first frame showing irreversible loss of balance followed by body descent or body-ground contact. For non-gait athletic actions involving aerial motion or landing phases (e.g., Diving, PoleVault, HighJump, LongJump, and TrampolineJumping), fall onset was defined as the first frame showing unsuccessful landing control, abnormal body orientation, failure of balance recovery after landing, or subsequent body-ground impact. For ball-control actions such as SoccerJuggling and VolleyballSpiking, fall onset was defined as the first frame indicating a visible breakdown of postural control leading to uncontrolled body descent or impact. These operational definitions were used during annotation to ensure consistency across different categories of athletic movement. Based on this onset frame, we defined the pre-fall interval as the temporal window immediately preceding the fall event.

In our implementation, frames within 30 frames before the fall onset were labeled as pre-fall risk frames ( $y_{t}=1$ ), corresponding to approximately 1 s under the standard video frame rate. Frames after the fall onset were treated as fall-event frames and were excluded from positive pre-fall supervision. Frames sampled from temporally stable movement periods that were sufficiently distant from the fall onset were labeled as non-risk frames ( $y_{t}=0$ ).

The 30-frame anticipation window was selected because biomechanical instability preceding a fall is often manifested through short-term transitional changes in posture and coordination, such as trunk imbalance, irregular lower-limb control, and unstable deceleration. A temporal window of approximately 1 s provides sufficient context to capture these observable instability patterns while remaining close enough to the actual fall event to support meaningful early-warning prediction.

The annotation process was conducted independently by two annotators with experience in sports movement analysis and pose-based video assessment. Each annotator identified the fall onset frame and assigned the corresponding pre-fall interval. Disagreements were resolved through discussion and secondary review. Inter-rater agreement was evaluated using Cohen’s kappa coefficient, yielding κ=0.86 , indicating strong agreement between annotators.

After annotation, the final subset contained 214 videos, 214 fall events, 42,657 total annotated frames, 6,420 pre-fall frames, 5,420 fall-event frames, and 30,817 non-risk frames. Fall-event frames were retained for dataset characterization but were not used as positive pre-fall supervision. The detailed subset composition and split information are summarized in Table 1. Representative category-level annotation criteria are provided in Table 2 to clarify how stable movement, pre-fall frames, fall onset, and fall-event frames were distinguished across gait-like, aerial, and sport-specific actions. The number of selected videos in each UCF101 category is summarized in Table 3.

**TABLE 1:** Composition of the manually constructed fall-related subset from UCF101.

Item	Value
Selected UCF101 action categories	11
Selected videos	214
Annotated fall events	214
Video duration range	2.1–7.8 s
Total annotated frames	42,657
Pre-fall frames ( $y_{t}=1$ )	6,420
Fall-event frames	5,420
Non-risk frames ( $y_{t}=0$ )	30,817
Pre-fall temporal window	30 frames ( ∼ 1 s)
Inter-rater agreement	κ=0.86
Training/validation/test split	150/21/43 videos
Split protocol	Video/event-level separation

**TABLE 2:** Representative annotation criteria for distinguishing stable movement, pre-fall frames, fall onset, and fall-event frames in selected sport categories. These examples were used to guide annotation consistency; they are described as skeleton-level or posture-level criteria rather than raw video-frame examples.

Category	Stable movement	Pre-fall frames	Fall onset	Fall-event frames
Basketball	Controlled running, turning, or landing with recoverable trunk and lower-limb alignment	Increasing trunk sway, unstable deceleration, asymmetric foot placement, or delayed postural recovery before descent	First frame showing irreversible balance loss followed by body descent or contact	Frames after onset showing body-ground contact or uncontrolled collapse
SkateBoarding	Balanced board motion with consistent pelvis-trunk alignment and controlled limb placement	Board-body separation, excessive trunk tilt, widened arm compensation, or failed recovery attempt	First frame where the body trajectory becomes unrecoverable relative to the board	Impact, sliding, or prolonged body-surface contact
Skiing	Continuous gliding or turning with recoverable knee, hip, and trunk control	Increasing lateral lean, ski divergence, knee collapse, or delayed balance recovery	First frame showing unrecoverable descent or fall toward the snow surface	Sliding or body-snow contact after loss of control
Diving	Intended aerial rotation or descent with controlled body orientation	Abnormal trunk orientation, poor landing preparation, or loss of body-line control before water entry	First frame showing unsuccessful landing or uncontrolled body orientation inconsistent with the intended movement phase	Post-onset frames in which the athlete remains in uncontrolled descent or impact state
TrampolineJumping	Cyclic bouncing with controlled vertical alignment and recoverable landing posture	Off-axis trunk rotation, unstable landing preparation, or failure to regain upright posture after bounce	First frame showing failed landing control or unrecoverable body orientation	Frames after onset showing collapse onto the trampoline surface or continued uncontrolled motion
HighJump	Approach, takeoff, or bar-clearance phase with expected sport-specific trunk inclination	Excessive trunk rotation or limb asymmetry beyond the expected movement phase, with poor landing preparation	First frame showing failed landing control or unrecoverable descent	Landing-collapse or uncontrolled body-surface contact
SoccerJuggling	Repeated ball-control motion with stable support-leg and trunk control	Support-leg instability, abrupt trunk compensation, or failed balance recovery during ball control	First frame showing loss of support control leading to uncontrolled descent	Frames after onset showing body-ground contact or continued collapse
VolleyballSpiking	Approach, jump, and arm swing with controlled landing preparation	Unstable landing posture, excessive lateral trunk displacement, or asymmetric lower-limb control after spike	First frame showing failed landing recovery or body descent	Post-onset frames showing fall, collapse, or body-surface contact

**TABLE 3:** Selected UCF101 action categories and number of videos included in the fall-related subset.

UCF101 category	Number of selected videos
Basketball	18
BasketballDunk	21
Diving	24
HighJump	19
LongJump	20
PoleVault	17
SkateBoarding	22
Skiing	18
SoccerJuggling	16
TrampolineJumping	21
VolleyballSpiking	18
Total	214

For model training and evaluation, the dataset was split at the video/event level into 70% training, 10% validation, and 20% testing subsets. No frames originating from the same continuous fall event were shared across different subsets, thereby preventing temporal leakage between training and evaluation data. Accordingly, the experimental findings should be interpreted as evidence of algorithmic feasibility on a manually annotated sports-motion subset, rather than as validation of a medical-grade fall-risk screening tool. The manually constructed labels indicate visually identifiable pre-fall intervals in sports videos, but they do not replace clinical fall-risk labels derived from prospective injury records, force-platform measurements, wearable sensors, or expert clinical assessment. External validation on real-world sports training videos, rehabilitation datasets, and multimodal biomechanical recordings will be required before the framework can be considered suitable for clinical or operational deployment.

**FIGURE 1:**
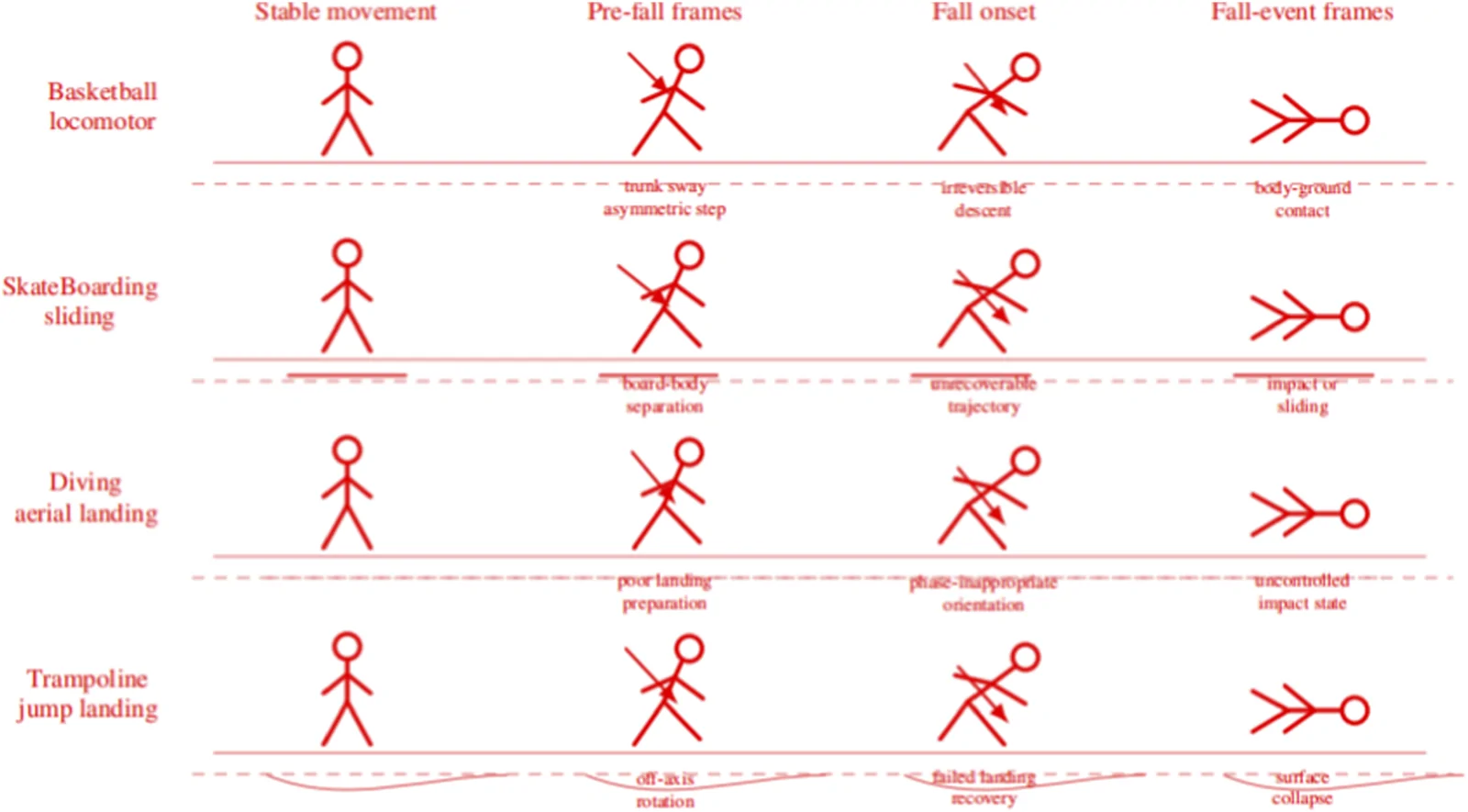
Representative schematic stick-figure sequences used to clarify the annotation logic for fall-related events Each rowillustrates one sport-motion context and cach column shows one annotation stage: stable movement, pre-fall frames, fall onset,and fall-event frames. These schematic examples are not original UCF101 frames; they visualize the posture-stage criteria usedtogether with Table 2.

To further reduce subjective interpretation of terms such as “unstable movement,” “failed landing,” and “loss of control,” we added representative schematic stick-figure sequences in Figure 1. These examples do not reproduce original UCF101 video frames. Instead, they provide visualized annotation logic for four representative sport-motion contexts by showing how annotators distinguished stable movement, pre-fall frames, fall onset, and fall-event frames. The examples should be read together with Table 2: the table defines the category-level criteria, while the figure illustrates the corresponding posture-stage transitions used during annotation.

Representative schematic stick-figure sequences used to clarify the annotation logic for fall-related events. Each row illustrates one sport-motion context and each column shows one annotation stage: stable movement, pre-fall frames, fall onset, and fall-event frames. These schematic examples are not original UCF101 frames; they visualize the posture-stage criteria used together with Table 2.

### Biomechanically constrained 3D skeleton refinement

Although recent advances in monocular 3D human pose estimation have improved the extraction of skeleton sequences from RGB videos, the resulting skeletons are still vulnerable to missing joints, temporal discontinuities, and jitter caused by occlusion, motion blur, and low-confidence detections. These issues are particularly problematic for pre-fall risk prediction, where subtle temporal changes in posture and coordination must be captured reliably.

It is important to clarify that RePoseNet is not designed as a standalone monocular 3D pose estimation model. Instead, it is a post-estimation skeleton refinement framework that operates on initially estimated 3D skeleton sequences produced by an existing pose estimator. Given an initial skeleton sequence $X\in R^{T\times J\times 3}$ , RePoseNet outputs a refined sequence $\hat{X}\in R^{T\times J\times 3}$ with improved temporal consistency and biomechanical plausibility.

**FIGURE 2:**
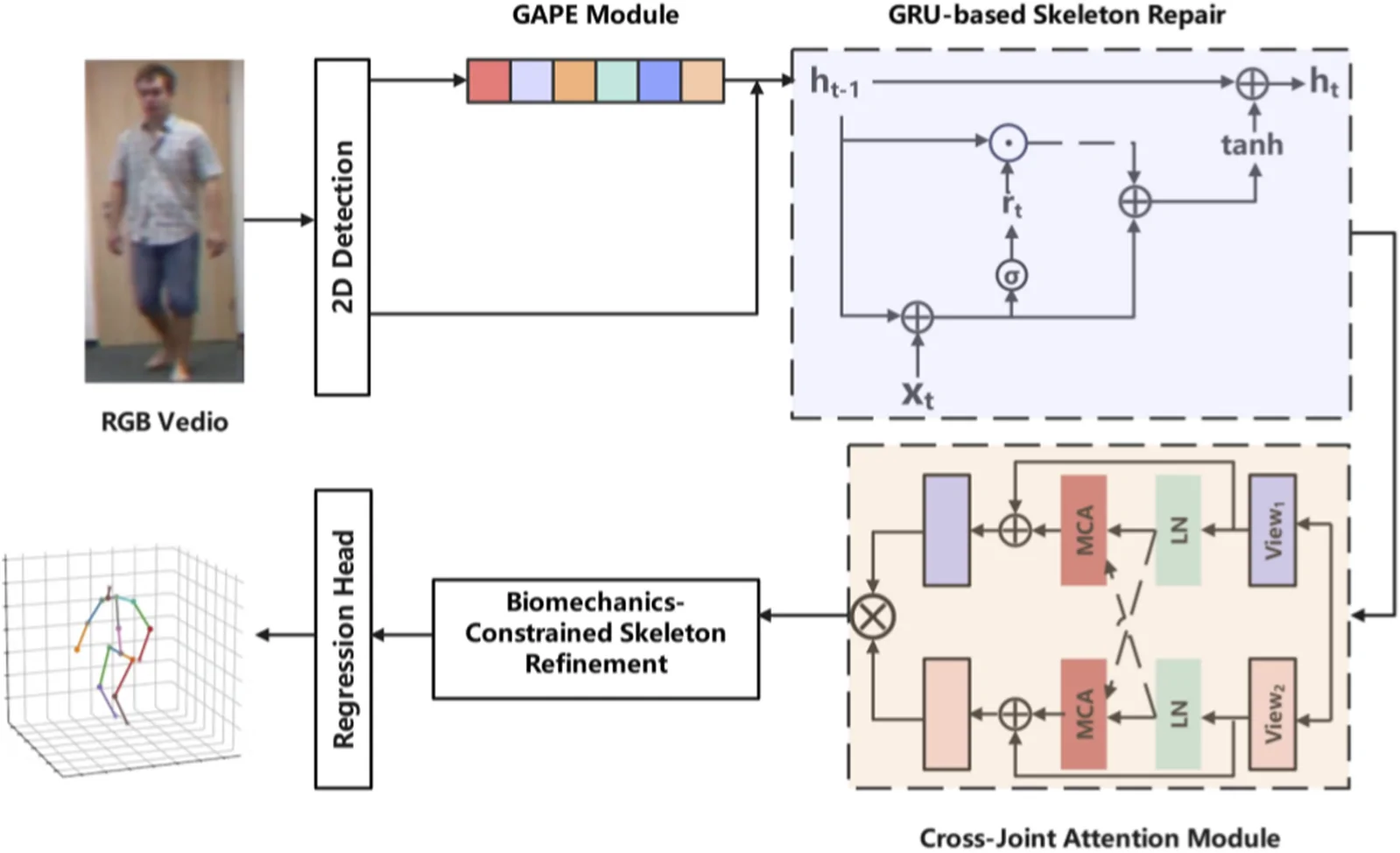
An overview of the proposed RePoseNet framework for biomechanically constrained 3D skeleton refinement. RePoseNet operates on initially estimated 3D skeleton sequences generated by a pretrained monocular pose estimation pipeline, rather than directly estimating 3D poses from raw RGB frames. The framework contains three main components: (1) the Gait-Aware Positional Encoding (GAPE) module, which encodes temporal gait-phase information; (2) the GRU-based Skeleton Repair module, which reconstructs missing or unreliable joint trajectories and improves temporal continuity; (3) the Cross-Joint Attention Module, which models inter-joint dependencies. The resulting skeleton sequence is further optimized by the Biomechanics-Constrained Skeleton Refinement module, which incorporates anatomical priors such as limb symmetry, limb-length consistency, joint-angle limits, and task-specific joint weighting. The output is a refined 3D skeleton sequence for downstream fall-risk prediction.

Unlike conventional pose-enhancement pipelines that independently apply temporal smoothing or spatial correction, RePoseNet is designed as a unified biomechanical refinement framework tailored for athletic-motion instability analysis. The novelty of RePoseNet lies not in the individual use of GRUs, attention mechanisms, or anatomical constraints themselves, but in their coordinated integration for instability-sensitive skeleton refinement. Specifically, the framework combines three tightly coupled components. First, the Gait-Aware Positional Encoding (GAPE) module injects motion-phase information into temporal representations, enabling the model to preserve rhythm or phase consistency during rapid athletic transitions. Second, the GRU-based repair module reconstructs corrupted trajectories while explicitly maintaining temporal continuity under unstable motion conditions. Third, the Cross-Joint Attention and biomechanical refinement modules jointly enforce inter-joint coordination and anatomical plausibility through symmetry preservation, limb-length regularization, and physiological angle constraints. This design differs fundamentally from generic pose denoising or sequence smoothing approaches because the refinement process is explicitly guided by biomechanical stability requirements relevant to pre-fall analysis, rather than only minimizing geometric reconstruction error. The overall architecture of RePoseNet is illustrated in Figure 2.

### Gait-Aware Positional Encoding (GAPE)

To capture individual differences in temporal movement patterns (e.g., step frequency, approach rhythm, or landing-recovery rhythm), we introduce a Gait-Aware Positional Encoding (GAPE) strategy. In this study, GAPE is used as a movement-phase encoding module rather than a walking-only descriptor. It modulates the input skeleton sequence with sinusoidal embeddings aligned to the subject’s inferred motion cycle when a periodic or quasi-periodic phase can be estimated.

For each time step $t\in \left[1,T\right]$ , we define a normalized movement phase $\phi_{t}\in \left[0,2\pi \right]$ based on estimated step or lower-limb periodicity (from knee or ankle vertical oscillations). The positional encoding vector $p_{t}\in R^{d}$ is defined as:

$$p_{t}^{\left(2i\right)}=\sin\left(\frac{\phi_{t}}{10000^{2i/d}}\right),p_{t}^{\left(2i+1\right)}=\cos\left(\frac{\phi_{t}}{10000^{2i/d}}\right)$$

Each joint’s 3D coordinate $x_{t,j}$ is embedded and augmented as:

$${\tilde{x}}_{t,j}=\text{MLP}\left(\left[x_{t,j};p_{t}\right]\right)$$

where $\left[\cdot ;\cdot \right]$ denotes concatenation. This enriched representation ${\tilde{x}}_{t,j}$ is then passed to the attention and GRU modules.

GAPE enhances temporal alignment and enables the network to adaptively model motion phases across subjects with diverse walking styles.

### Temporal enhancement via GRU-based skeleton repair

In real-world scenarios, 3D pose estimation outputs frequently suffer from missing joints, detection noise, and temporal discontinuity—especially under conditions of occlusion, low contrast, or rapid motion. To address this, we design a temporal enhancement module that leverages gated recurrent units (GRUs) to reconstruct corrupted or missing joint trajectories by modeling temporal dependencies. This module constitutes the first stage of the proposed RePoseNet framework.

Let the initial estimated 3D skeleton sequence be denoted as $X\in R^{T\times J\times 3}$ , where T is the total number of frames, J is the number of joints, and each joint has a 3D coordinate $\left(x,y,z\right)$ . Due to occlusion or low confidence, certain joints may be missing (set to zero) or unreliable. We define a binary mask $M\in {\left\{0,1\right\}}^{T\times J}$ such that $M_{t,j}=0$ indicates a corrupted joint $x_{t,j}$ at frame t and joint index j .

To reconstruct these corrupted joints, we model the trajectory of each joint j independently as a temporal sequence $\left\{x_{1,j},x_{2,j},\ldots ,x_{T,j}\right\}$ . This sequence is encoded using a GRU network as follows:

$$h_{t}^{j}=\text{GRU}\left(x_{t-1,j},h_{t-1}^{j}\right),{\tilde{x}}_{t,j}=\text{MLP}\left(h_{t}^{j}\right)$$

where $h_{t}^{j}$ denotes the hidden state of joint j at time t , and ${\tilde{x}}_{t,j}\in R^{3}$ is the reconstructed joint position. For corrupted entries ( $M_{t,j}=0$ ), the reconstructed joint replaces the original estimate:

$$x_{t,j}^{\text{out}}=\left(1-M_{t,j}\right)\cdot {\tilde{x}}_{t,j}+M_{t,j}\cdot x_{t,j}$$

This ensures that reliable joints remain unchanged, while corrupted ones are substituted with learned reconstructions.

The masking procedure was applied exclusively to pseudo-3D skeleton coordinates rather than RGB frames or 2D image observations. Specifically, a predefined number of joints were randomly selected and replaced with zero-valued coordinates within individual frames. The masking process was performed independently for each frame and did not explicitly model temporally persistent occlusions. As a result, neighboring frames often retained information about joints that were missing in the current frame, allowing the model to use temporal interpolation and learned motion priors during reconstruction. Therefore, the masking experiment should be interpreted as a controlled skeleton-level corruption and missing-joint recovery protocol rather than a simulation of image-level occlusion, motion blur, detector failure, or full visual pose-estimation robustness. The objective is to minimize the reconstruction error between predicted and original joint positions:

$$L_{\text{GRU}}=\frac{1}{\left|\Omega \right|}\sum_{\left(t,j\right)\in \Omega }∥{\tilde{x}}_{t,j}-x_{t,j}{∥}_{2}^{2}$$

where Ω is the set of artificially masked joints during training. Additionally, to suppress temporal jitter and enforce local smoothness, we introduce a temporal consistency loss over successive frames:

$$L_{\text{smooth}}=\frac{1}{T-1}\sum_{t=1}^{T-1}\sum_{j=1}^{J}∥x_{t+1,j}^{o\text{ut}}-x_{t,j}^{\text{out}}{∥}_{2}^{2}$$

The overall loss for the temporal enhancement module is defined as:

$$L_{\text{temporal}}=L_{\text{GRU}}+\lambda_{\text{smooth}}\cdot L_{\text{smooth}}$$

where $\lambda_{\text{smooth}}$ controls the trade-off between reconstruction fidelity and temporal consistency. The enhanced skeleton sequence $X^{\text{out}}$ is then passed to the second module of RePoseNet for biomechanical refinement.

### Cross-joint attention module

Although modeling joint trajectories individually allows for effective noise reduction, it overlooks the inherent spatial dependencies among body joints. To better capture structural coordination (e.g., simultaneous movement of both knees), we incorporate a Cross-Joint Attention Module that enables each joint to attend to all others within the same frame.

Given the input skeleton sequence $X\in R^{T\times J\times 3}$ , we project each joint at time t into a latent embedding:

$$q_{t,j}=W_{Q}x_{t,j},k_{t,j}=W_{K}x_{t,j},v_{t,j}=W_{V}x_{t,j}$$

where $W_{Q}$ , $W_{K}$ , and $W_{V}\in R^{d\times 3}$ are learnable linear projections. The attention-weighted joint representation ${\hat{x}}_{t,j}$ is computed as:

$${\hat{x}}_{t,j}=\sum_{j^{\prime }=1}^{J}\alpha_{j,j^{\prime }}\cdot v_{t,j^{\prime }},\alpha_{j,j^{\prime }}=\frac{\exp\left(q_{t,j}^{⊤}k_{t,j^{\prime }}\right)}{\sum_{k=1}^{J}\exp\left(q_{t,j}^{⊤}k_{t,k}\right)}$$

This joint-aware embedding ${\hat{x}}_{t,j}$ replaces $x_{t,j}$ as input to the subsequent GRU encoder:

$$h_{t}^{j}=\text{GRU}\left({\hat{x}}_{t-1,j},h_{t-1}^{j}\right)$$

By explicitly modeling inter-joint dependencies, this module improves spatial coherence and enhances the anatomical plausibility of reconstructed skeletons.

### Biomechanics-constrained skeleton refinement

Although GRU-based temporal enhancement reduces missing joints and improves sequence continuity, the resulting skeletons may still suffer from anatomically implausible configurations, such as irregular limb lengths, asymmetric postures, or unnatural joint angles. These structural errors are particularly detrimental in fall-risk assessment tasks, where anatomical consistency and motion integrity are critical for accurate recognition of pre-fall anomalies.

To address this issue, we introduce a biomechanical-constrained refinement module that further optimizes the enhanced skeleton sequence $X^{\text{out}}\in R^{T\times J\times 3}$ by enforcing anatomical priors and task-guided correction. The refinement process involves two key components: (1) structural consistency constraints that align the output with human kinematic norms; and (2) task-aware weighting that emphasizes stability-critical joints (hips, knees, trunk).

We first enforce limb-length consistency to ensure that relative distances between anatomically linked joints remain stable throughout the sequence. Let E be the set of connected joint pairs (limbs), and let $d_{ij}^{t}=∥x_{t,i}^{\text{out}}-x_{t,j}^{\text{out}}{∥}_{2}$ be the length of limb $\left(i,j\right)$ at time t . The target limb length ${\bar{d}}_{ij}$ is estimated as the temporal mean over the sequence. The limb consistency loss is defined as:

$$L_{\text{limb}}=\frac{1}{T\cdot \left|E\right|}\sum_{t=1}^{T}\sum_{\left(i,j\right)\in E}{\left(d_{ij}^{t}-{\bar{d}}_{ij}\right)}^{2}$$

To preserve symmetry, we further constrain the left-right joint pairs (e.g., left/right shoulders, knees, ankles) to maintain mirror distances with respect to the body centerline. Let $\left(i_{L},i_{R}\right)$ be a symmetric joint pair and $c_{t}$ denote the mid-point between shoulders or hips. The symmetry loss is:

$$L_{\text{sym}}=\frac{1}{T\cdot \left|P\right|}\sum_{t=1}^{T}\sum_{\left(i_{L},i_{R}\right)\in P}\left|∥x_{t,i_{L}}-c_{t}{∥}_{2}-∥x_{t,i_{R}}-c_{t}{∥}_{2}\right|$$

Additionally, we regularize joint angles to remain within physiological ranges. For each triple of joints $\left(i,j,k\right)$ forming an angle at j , we compute the joint angle $\theta_{ijk}^{t}$ and enforce range constraints $\left[\theta_{\min},\theta_{\max}\right]$ :

$$L_{\text{angle}}=\sum_{t=1}^{T}\sum_{\left(i,j,k\right)}\max\left(0,\theta_{ijk}^{t}-\theta_{\max}\right)+\max\left(0,\theta_{\min}-\theta_{ijk}^{t}\right)$$

We introduce task-aware weighting to guide the refinement process toward joints that are critical for stability assessment. Let $w_{j}$ be a joint-specific weight, where higher values are assigned to hips, knees, and trunk joints based on their relevance to fall-risk prediction. The weighted reconstruction loss is:

$$L_{\text{task}}=\frac{1}{T\cdot J}\sum_{t=1}^{T}\sum_{j=1}^{J}w_{j}\cdot ∥{\hat{x}}_{t,j}-x_{t,j}^{\text{out}}{∥}_{2}^{2}$$

In our implementation, higher task-aware weights were assigned to stability-critical joints based on their biomechanical relevance to postural control. Specifically, trunk, pelvis, hip, and knee joints were assigned a weight of 2.0, while other joints were assigned a default weight of 1.0. This weighting strategy encourages the refinement process to prioritize anatomically and functionally important regions for fall-risk prediction.

The overall loss for the biomechanical refinement module is:

$$L_{\text{refine}}=\lambda_{1}L_{\text{limb}}+\lambda_{2}L_{\text{sym}}+\lambda_{3}L_{\text{angle}}+\lambda_{4}L_{\text{task}}$$

where $\lambda_{1}$ through $\lambda_{4}$ are tunable hyperparameters. The final refined skeleton sequence $\hat{X}=\left\{{\hat{x}}_{t,j}\right\}$ is used for stability feature extraction and risk classification in the subsequent modeling stages.

The final training objective for RePoseNet combines temporal reconstruction and biomechanical refinement losses:

$$L_{\text{total}}=L_{\text{temporal}}+L_{\text{refine}}.$$

This unified objective jointly optimizes temporal continuity, anatomical plausibility, and stability-aware joint consistency within a single end-to-end framework. From a representation-learning perspective, these biomechanical regularization terms constrain the feasible optimization space of the network and bias the learned latent motion representations toward physically consistent coordination patterns. As a result, the model learns not only temporally smooth trajectories but also anatomically meaningful motion structures that are more robust under occlusion, rapid movement, and pose corruption.

The proposed RePoseNet framework addresses two critical challenges in pseudo-3D skeleton-based pre-fall analysis: temporal corruption and biomechanical inconsistency. By integrating a GRU-based skeleton repair module and a biomechanics-constrained refinement module, RePoseNet improves motion continuity and anatomical plausibility in reconstructed skeleton sequences. The temporal module learns to impute corrupted or missing joints by exploiting temporal dependencies, while the refinement module enforces human kinematic constraints and task-specific anatomical consistency. Together, these components provide more stable pseudo-3D skeleton inputs for downstream movement-stability modeling and early fall-risk prediction.

### Fall-risk prediction via dynamic stability-aware modeling

While enhanced 3D skeleton sequences provide a temporally coherent and anatomically plausible representation of human motion, detecting imminent fall risks remains a formidable challenge. Pre-fall behaviors often manifest as subtle and transient kinematic anomalies—such as abrupt changes in movement rhythm, deviations in trunk posture, or asymmetrical lower-limb coordination—that are difficult to capture using only raw joint coordinates.

To address this, we introduce StaFallNet, a stability-aware dynamic graph framework specifically designed for pre-fall risk modeling in athletic motion. Although graph convolution and temporal modeling have been widely explored in skeleton-based action analysis, existing approaches generally rely on fixed anatomical graphs and purely data-driven feature propagation.

The key innovation of StaFallNet is that biomechanical instability indicators are directly incorporated into the graph topology adaptation process. Instead of treating all inter-joint connections as static or uniformly important, the proposed model dynamically modulates graph connectivity according to stability-related motion cues, enabling the network to focus on transient coordination disruptions that precede falls.

This design allows the model to move beyond conventional action-recognition-oriented graph learning and explicitly model instability propagation patterns associated with loss of postural control.

1. The Stability Feature Extraction Module computes short-term kinematic indicators—such as step frequency variation rate (SFVR), trunk leaning angle (TLA), and stride variability (SV)—that reflect momentary movement instability and serve as auxiliary inputs to StaFallNet.
2. The Dynamic Graph Topology Learning Module constructs adaptive inter-joint graphs that evolve over time, enabling the network to focus on transient coordination patterns between key stability joints (e.g., hips, knees, trunk) that often precede a fall. The dynamic topology is modulated by the extracted biomechanical features to reflect context-aware dependencies.
3. The Fall-Risk Classification Head receives the aggregated node representations through an attention mechanism and outputs a continuous fall-risk score for each frame, supporting both real-time inference and multi-window future forecasting for early intervention.

Together, these components allow the system to reason not only about pose geometry but also about biomechanical integrity and coordination dynamics—thus enabling more accurate and interpretable fall-risk prediction.

### Stability feature extraction module

To enhance the interpretability and robustness of fall-risk prediction, we extract a set of biomechanically grounded kinematic features from the 3D skeleton sequence. These features are designed to capture critical patterns of movement instability and postural-control deterioration that may emerge before loss of balance in athletic actions. Specifically, we compute three frame-wise indicators: step frequency variation rate (SFVR), trunk leaning angle (TLA), and stride variability (SV). These handcrafted stability features were provided only to StaFallNet and were not supplied to the generic CNN, Transformer, PoseFormer, or DeGCN comparison baselines. Therefore, the StaFallNet comparison should be interpreted as evaluating the combined contribution of the feature-engineering pipeline, feature-fusion strategy, and dynamic graph architecture, rather than isolating the neural architecture alone.

The selected stability features are designed to capture intrinsic biomechanical instability mechanisms commonly observed in athletic movements. Step Frequency Variation Rate (SFVR) reflects abrupt disruption of gait-like or task-specific movement rhythm, which may occur during unstable deceleration, approach steps, landing preparation, or recovery phases. Trunk Leaning Angle (TLA) characterizes anterior, posterior, or lateral trunk displacement, which is strongly associated with loss of postural control and center-of-mass instability. Stride Variability (SV) measures inconsistency in lower-limb coordination and temporal movement symmetry; for non-gait or aerial actions, it is interpreted as a local lower-limb displacement variability measure rather than a conventional clinical stride descriptor. Together, these features allow the model to identify internally generated instability patterns related to impaired coordination and postural regulation, rather than unpredictable externally induced falls.

The step frequency variation rate (SFVR) characterizes the temporal fluctuation of locomotor or movement rhythm. Although originally motivated by gait analysis, the measure can also reflect instability-related disruptions in repetitive athletic movement cycles, including approach gait, landing preparation, directional transition, and lower-limb coordination. In gait-like phases, step frequency exhibits regular oscillation, whereas abrupt shifts often indicate motor instability. In aerial or non-gait actions, the same signal is used only when a meaningful approach, landing, or recovery segment exists; otherwise it is interpreted as a local movement-rhythm cue rather than a walking-specific gait variable. We approximate this frequency by analyzing the temporal signal formed by the Euclidean distance between the left and right ankle joints:

$$d_{\text{ankle}}^{t}={∥{\hat{x}}_{t,{\text{ankle}}_{L}}-{\hat{x}}_{t,{a\text{nkle}}_{R}}∥}_{2},$$

where ${\hat{x}}_{t,j}\in R^{3}$ is the position of joint j at frame t . Using a short-time Fourier transform (STFT) over a local temporal window $W_{t}$ , we extract the dominant movement frequency $f_{t}$ . The SFVR is then defined as the normalized change in this frequency across consecutive frames:

$${\text{SFVR}}_{t}=\frac{\left|f_{t}-f_{t-1}\right|}{f_{t-1}+\epsilon },$$

where ϵ is a small positive constant ensuring numerical stability. Larger values of SFVR imply abrupt rhythm transitions, often linked to deteriorated control.

In our implementation, the STFT was computed using a 16-frame sliding window with 50% overlap. The dominant frequency component within each window was selected as the estimated movement-rhythm frequency.

In addition to rhythm irregularities, postural instability is another hallmark of fall risk. The trunk leaning angle (TLA) quantifies the degree of anterior, posterior, or lateral inclination of the upper body, which can signal a loss of balance. Because some sport phases such as HighJump or Diving naturally involve large trunk inclination, TLA was interpreted relative to the annotated movement phase rather than treated as an abnormality by itself. We define the trunk orientation vector as the displacement from the hip center to the neck joint:

$$v_{\text{trunk}}^{t}={\hat{x}}_{t,\text{neck}}-{\hat{x}}_{t,{\text{hip}}_{\text{center}}},$$

and compare it with the global vertical direction $v_{\text{up}}={\left[0,1,0\right]}^{⊤}$ using the cosine of their angle:

$${\text{TLA}}_{t}=\arccos\left(\frac{v_{\text{trunk}}^{t}\cdot v_{\text{up}}}{∥v_{\text{trunk}}^{t}∥\cdot ∥v_{\text{up}}∥}\right).$$

Excessively large or phase-inappropriate values of TLA reflect trunk displacement that may move the center of mass outside the recoverable support region and increase the likelihood of a fall.

Finally, stride variability (SV) measures the irregularity of lower-limb displacement across time. It is computed by first estimating the horizontal displacement interval of a single leg—e.g., the left ankle—as:

$$s_{t}={∥{\hat{x}}_{t,{\text{ankle}}_{L}}^{xy}-{\hat{x}}_{t-\delta ,{\text{ankle}}_{L}}^{xy}∥}_{2},$$

where ${\hat{x}}^{xy}$ denotes the $\left(x,y\right)$ projection of the joint position and δ is a fixed stride interval (e.g., 10 frames). The variance of stride lengths within a local window $W_{t}$ is then calculated as:

$${\text{SV}}_{t}=\frac{1}{\left|W_{t}\right|}\sum_{t^{\prime }\in W_{t}}{\left(s_{t^{\prime }}-{\bar{s}}_{t}\right)}^{2},{\bar{s}}_{t}=\frac{1}{\left|W_{t}\right|}\sum_{t^{\prime }\in W_{t}}s_{t^{\prime }}.$$

Higher SV values indicate inconsistent lower-limb motion or weakened neuromuscular coordination, which may be predictive of reduced stability. In non-gait or aerial actions, SV was computed over the approach, landing, or recovery phase when such a phase was present, rather than over the entire airborne interval.

We concatenate the three features into a temporal sequence $S={\left\{s_{t}\right\}}_{t=1}^{T}$ , where $s_{t}=\left[{\text{SFVR}}_{t},{\text{TLA}}_{t},{\text{SV}}_{t}\right]\in R^{d_{s}}$ and $d_{s}=3$ . This auxiliary feature sequence is encoded by a temporal MLP or GRU encoder and integrated into the graph-based prediction pipeline through concatenation or attention-based fusion, enabling the model to jointly reason over spatial kinematics and dynamic stability cues.

From a feature fusion perspective, the proposed StaFallNet framework integrates heterogeneous biomechanical representations at multiple levels. Specifically, refined spatial skeleton representations, temporal stability descriptors (SFVR, TLA, and SV), and dynamically learned inter-joint coordination patterns are jointly fused within the graph-based prediction pipeline. Rather than relying solely on raw joint trajectories, the proposed framework combines explicit biomechanical indicators with data-driven spatial-temporal representations, enabling complementary information integration for instability modeling.

This design is conceptually related to recent movement-analysis studies emphasizing structured feature fusion strategies for improving both predictive performance and interpretability in biomechanical assessment tasks (Zhou et al., 2026). In particular, feature-level integration of kinematic descriptors and stability-aware representations has been shown to enhance robustness under complex movement conditions. Inspired by this perspective, StaFallNet incorporates biomechanical stability cues directly into graph topology adaptation and attention-based feature aggregation, allowing the model to capture instability-related coordination changes more effectively.

### Dynamic graph topology learning module

Most existing skeleton-based action recognition models use fixed human joint connectivity graphs derived from anatomical topology, which fail to capture the dynamic co-movement patterns that emerge during movement instability. In the context of fall-risk prediction, this rigidity prevents the model from adapting to subtle, yet informative, shifts in inter-joint coordination—such as asynchronous leg movements or disproportionate trunk oscillations—that often manifest as early warning signs of a fall.

To overcome this limitation, we propose a dynamic graph topology learning module that constructs a frame-wise, context-aware adjacency matrix $A_{t}\in R^{J\times J}$ based on both the spatial configuration of the joints and the stability feature sequence $S={\left\{s_{t}\right\}}_{t=1}^{T}$ . This module enables the model to emphasize physiologically relevant interactions (e.g., knee–trunk synergy) while suppressing noisy or irrelevant connections in unstable movement episodes.

Let $\hat{X}=\left\{{\hat{x}}_{t,j}\right\}$ denote the refined skeleton sequence output from RePoseNet, where each frame t is represented by a set of J joints in $R^{3}$ . For each frame t , we compute the pairwise geometric distances:

$$D_{t}\left(i,j\right)={∥{\hat{x}}_{t,i}-{\hat{x}}_{t,j}∥}_{2},$$

and define a raw spatial affinity matrix via a Gaussian kernel:

$$A_{t}^{\text{geo}}\left(i,j\right)=\exp\left(-\frac{D_{t}{\left(i,j\right)}^{2}}{\sigma^{2}}\right),$$

where σ is a scaling factor controlling spatial locality.

To incorporate dynamic stability information, we further compute an attention-based modulation over the raw adjacency. Specifically, we pass the stability vector $s_{t}\in R^{d_{s}}$ through a two-layer perceptron to produce joint-level importance weights $w_{t}\in R^{J}$ :

$$w_{t}=\text{Softmax}\left(\text{MLP}\left(s_{t}\right)\right)=\text{Softmax}\left(W_{2}\cdot \text{ReLU}\left(W_{1}s_{t}+b_{1}\right)+b_{2}\right),$$

where $w_{t}\left(j\right)$ indicates the relevance of joint j under current stability conditions (e.g., hips and trunk are emphasized when SFVR or TLA are high). We then compute the final dynamic adjacency matrix by weighting the spatial affinities:

$$A_{t}\left(i,j\right)=w_{t}\left(i\right)\cdot A_{t}^{\text{geo}}\left(i,j\right)\cdot w_{t}\left(j\right),$$

which ensures that more attention is allocated to interactions involving stability-critical joints.

To improve numerical stability and prevent excessively dense graph connections, the dynamic adjacency matrix was further row-normalized after feature modulation:

$${\tilde{A}}_{t}\left(i,j\right)=\frac{A_{t}\left(i,j\right)}{\sum_{k=1}^{J}A_{t}\left(i,k\right)+\epsilon },$$

where ϵ is a small constant for numerical stability.

In practice, only the top- K strongest connections for each joint were retained to suppress noisy long-range dependencies. In our implementation, K=5 was used for all experiments. The Gaussian kernel parameter σ was empirically set to 0.8 based on validation performance.

The resulting sequence of dynamic graphs ${\left\{A_{t}\right\}}_{t=1}^{T}$ , along with node features $\left\{{\hat{x}}_{t,j}\right\}$ , is processed by a spatial-temporal graph convolutional network (ST-GCN or DeGCN) defined as:

$$h_{t,j}^{\left(l+1\right)}=\sigma \left(\sum_{k=1}^{J}A_{t}\left(j,k\right)\cdot W^{\left(l\right)}h_{t,k}^{\left(l\right)}+b^{\left(l\right)}\right),$$

where $h_{t,j}^{\left(l\right)}$ is the node embedding at layer l , and $\sigma \left(\cdot \right)$ is a non-linear activation such as ReLU. Temporal modeling is handled via adjacent-frame connections or interleaved GRU layers.

Unlike traditional fixed-graph ST-GCNs, our module dynamically modulates the adjacency structure at each frame, allowing the model to adaptively capture evolving inter-joint relationships indicative of biomechanical instability. This enables more precise modeling of compound pre-fall patterns—such as “knee buckling followed by forward trunk lean”—which may not be explicit in static anatomical graphs.

To encourage stable learning, we regularize the dynamic adjacency matrices with a sparsity constraint:

$$L_{\text{sparse}}=\frac{1}{T}\sum_{t=1}^{T}\sum_{i\neq j}A_{t}\left(i,j\right),$$

which penalizes overly dense graphs and promotes physiologically plausible connectivity patterns.

### Fall-risk classification head

Following the extraction of dynamic graph representations from the previous module, we integrate the encoded spatiotemporal embeddings to generate a fall-risk score for each frame, indicating the likelihood that the current posture and motion pattern represent a pre-fall anomaly. This prediction head serves as the final stage of our framework and transforms the learned joint-level features into a clinically meaningful signal.

Let $H\in R^{T\times J\times d}$ denote the spatiotemporal feature tensor output by the final layer of the graph convolutional network, where T is the number of frames, J is the number of joints, and d is the feature dimension of each joint embedding. To derive a compact frame-level representation, we apply joint-wise attention pooling to each frame, enabling the model to emphasize stability-critical joints (such as hips, knees, and trunk). Specifically, for each frame t , we compute a weighted aggregation of joint embeddings:

$$z_{t}=\sum_{j=1}^{J}w_{t}\left(j\right)\cdot h_{t,j}$$

where $h_{t,j}\in R^{d}$ represents the feature vector of joint j at time t , and $w_{t}\left(j\right)$ is the attention weight indicating the importance of joint j for stability-aware fall-risk assessment.

The resulting temporal embedding sequence ${\left\{z_{t}\right\}}_{t=1}^{T}$ is passed through a lightweight temporal aggregator, such as a GRU or temporal convolutional layer, to capture higher-order dependencies and long-range instability cues:

$${\hat{z}}_{t}=\text{GRU}\left(z_{t-1},{\hat{z}}_{t-1}\right)$$

Each hidden representation ${\hat{z}}_{t}$ is then mapped to a scalar risk score ${\hat{y}}_{t}\in \left[0,1\right]$ using a fully connected layer with sigmoid activation:

$${\hat{y}}_{t}=\sigma \left(W_{f}\cdot {\hat{z}}_{t}+b_{f}\right)$$

where ${\hat{y}}_{t}$ represents the predicted fall-risk probability at frame t , and $\sigma \left(\cdot \right)$ denotes the sigmoid function. This output allows real-time monitoring of risk evolution across the sequence and facilitates early-warning interventions before a fall occurs.

To train the classification head, we use a binary cross-entropy loss over all labeled frames:

$$L_{\text{cls}}=-\frac{1}{T}\sum_{t=1}^{T}\left(y_{t}\cdot \log{\hat{y}}_{t}+\left(1-y_{t}\right)\cdot \log\left(1-{\hat{y}}_{t}\right)\right)$$

where $y_{t}\in \left\{0,1\right\}$ is the manually annotated frame-level label. Specifically, $y_{t}=1$ denotes a pre-fall risk frame within the predefined temporal window before fall onset, whereas $y_{t}=0$ denotes a non-risk frame sampled from stable movement periods. For clinical deployment, the system can output an average risk score over a sliding time window (e.g., 1–2 s), or trigger an alert when ${\hat{y}}_{t}$ exceeds a predefined threshold.

This classification head completes the end-to-end architecture of our proposed system, enabling the model to not only enhance and refine skeleton input but also to interpret the resulting dynamics in terms of actionable fall-risk estimation. The combination of stability-aware features, dynamic topology modeling, and temporal decoding forms a coherent pipeline that addresses the critical need for interpretable and reliable pre-fall detection in real-world scenarios.

### Artificial joint corruption protocol

To evaluate the missing-joint recovery capability of RePoseNet under controlled conditions, we conducted an artificial joint corruption experiment.

For each skeleton sequence, a predefined number of joints were randomly selected and replaced with missing values. Corruption levels of 4, 8, 12, and 16 joints were considered. Missing joints were encoded as zero-valued 3D coordinates, and a binary visibility mask M was used to identify whether each joint was observed or corrupted.

The corruption was applied directly to pseudo-3D skeleton coordinates after pose estimation rather than to RGB frames or 2D detections. Masked joints were independently sampled for each frame and therefore did not necessarily remain missing across consecutive frames. Consequently, the model could exploit adjacent frames in which the same anatomical joint remained visible, making the experiment a test of temporal skeleton interpolation and reconstruction consistency rather than recovery from complete information loss.

The purpose of this experiment was to evaluate the ability of RePoseNet to recover incomplete skeleton observations and maintain temporal consistency under controlled skeleton-level corruption. Importantly, the experiment does not evaluate robustness of the complete visual pose-estimation pipeline to image-level occlusion, motion blur, camera artifacts, or detector failure.

The most severe setting (16 missing joints in the COCO-17 skeleton format) represents an intentionally extreme corruption scenario in which only one original joint remains observed in a given frame. This setting should be interpreted as a stress test of temporal interpolation and learned skeleton priors, not as evidence that RePoseNet can accurately recover body pose under ordinary real-world near-complete visual occlusion.

## Evaluation metrics

### Evaluation of RePoseNet

To evaluate the effectiveness of RePoseNet as a skeleton refinement module, we report Mean Per Joint Position Error (MPJPE) and Percentage of Correct Keypoints (PCK). However, we clarify that these metrics are computed with respect to pseudo-3D reference skeletons generated by the pretrained monocular pose estimation pipeline, because UCF101 does not provide true 3D joint annotations.

Formally, let ${\hat{p}}_{t,j}\in R^{3}$ denote the refined joint coordinate predicted by RePoseNet, and let $p_{t,j}^{ref}\in R^{3}$ denote the corresponding pseudo-3D reference joint coordinate generated by the pretrained estimator. MPJPE is computed as:

$${\text{MPJPE}}_{ref}=\frac{1}{T\times J}\sum_{t=1}^{T}\sum_{j=1}^{J}{∥{\hat{p}}_{t,j}-p_{t,j}^{ref}∥}_{2},$$

where T is the sequence length and J is the number of joints. A lower ${\text{MPJPE}}_{ref}$ indicates that the refined skeleton remains closer to the reference pose while improving temporal and biomechanical consistency.

PCK is computed as the proportion of joints whose refined positions lie within a predefined distance threshold δ from the pseudo-3D reference skeleton:

$${\text{PCK}}_{ref}@\delta =\frac{1}{T\times J}\sum_{t=1}^{T}\sum_{j=1}^{J}I\left({∥{\hat{p}}_{t,j}-p_{t,j}^{ref}∥}_{2}<\delta \right),$$

where $I\left(\cdot \right)$ is the indicator function.

Accordingly, MPJPE and PCK are used only as reference-consistency metrics under the absence of motion-capture reference trajectory generated by the pretrained pose estimator.

Because the pseudo-3D reference skeletons are also the initial inputs to RePoseNet, improvements in MPJPE or PCK should not be interpreted as direct evidence of improved 3D pose accuracy.

Instead, these metrics are intended to evaluate whether the refinement process preserves structural consistency while recovering corrupted joints and reducing temporal instability.

The practical effectiveness of RePoseNet is therefore assessed primarily through downstream fall-risk prediction performance and ablation studies within the complete StaFallNet pipeline.

### Evaluation of stability-aware fall-risk modeling

To comprehensively evaluate the proposed fall-risk prediction framework, we adopt a suite of standard classification metrics that jointly capture prediction correctness, sensitivity, specificity, and confidence calibration. These include accuracy, precision, recall, F1-score, and mean Average Precision (mAP), each providing complementary insights into the model’s performance in detecting pre-fall states. All classification metrics were computed using the manually constructed binary frame-level labels described in the label construction protocol. Positive samples correspond to pre-fall frames before fall onset, rather than frames after the fall had already occurred.

Formally, let $y_{t}\in \left\{0,1\right\}$ be the ground-truth label and ${\hat{y}}_{t}\in \left\{0,1\right\}$ be the predicted label at time step t , where 1 indicates a pre-fall risk state. Based on the counts of true positives (TP), false positives (FP), true negatives (TN), and false negatives (FN), the overall classification accuracy is defined as the ratio of correctly predicted samples:

$$\text{Accuracy}=\frac{\text{TP}+\text{TN}}{\text{TP}+\text{FP}+\text{TN}+\text{FN}}.$$

To assess the model’s ability to precisely identify fall-risk frames while minimizing false alarms, we further compute precision and recall, defined respectively as:

$$\text{Precision}=\frac{\text{TP}}{\text{TP}+\text{FP}},\text{Recall}=\frac{\text{TP}}{\text{TP}+\text{FN}}.$$

The F1-score, as the harmonic mean of precision and recall, balances these two metrics to reflect the trade-off between type I and type II errors:

$$F1‐\text{score}=2\cdot \frac{\text{Precision}\cdot \text{Recall}}{\text{Precision}+\text{Recall}}.$$

In addition to threshold-based metrics, we also compute the mean Average Precision (mAP), which evaluates the ranking quality of frame-level risk scores ${\hat{y}}_{t}\in \left[0,1\right]$ across varying thresholds. Given the precision–recall curve $\text{Prec}\left(\tau \right)$ and $\text{Rec}\left(\tau \right)$ parameterized by decision threshold τ , the average precision (AP) is defined as the area under the precision–recall curve:

$$\text{AP}=\int_{0}^{1}\text{Prec}\left({\text{Rec}}^{-1}\left(r\right)\right)\;dr,\text{mAP}=\frac{1}{C}\sum_{c=1}^{C}{\text{AP}}_{c},$$

where C=2 in our binary classification setup. This metric captures the model’s ability to assign high risk scores to truly risky frames, ensuring reliable early warnings in safety-critical deployment scenarios.

### Baseline configuration

For fair comparison, all RePoseNet baselines were evaluated under the same skeleton-refinement protocol. The compared baselines include Temporal CNN (TCNN), MHFormer (Li et al., 2022), STCFormer (Tang et al., 2023), and DTF (Ghafoor et al., 2024).

TCNN is an author-implemented simple temporal convolutional reconstruction baseline rather than a previously published model. It uses three one-dimensional temporal convolution layers with kernel size 3, hidden channel dimensions of 64, 128, and 128, ReLU activations, and a linear output projection that restores the J×3 pseudo-3D skeleton coordinates.

MHFormer, STCFormer, and DTF were originally proposed mainly for pose estimation or spatiotemporal pose modeling. In this study, they were adapted to the same 3D-to-3D pseudo-skeleton reconstruction task as RePoseNet. The original 2D-to-3D lifting or task-specific prediction heads were replaced with 3D reconstruction heads that output complete pseudo-3D skeleton sequences. All adapted baselines were trained from scratch on the same training split used by RePoseNet; no pretrained weights from the original pose-estimation tasks were used.

For every baseline, the input consisted of corrupted pseudo-3D skeleton coordinates together with the same binary visibility mask M used in the artificial corruption protocol. Missing joints were represented by zero-valued coordinates. The output of each model was a reconstructed skeleton sequence with the same dimensionality as the pseudo-reference skeleton. The reconstruction objective was computed over all joints to preserve whole-skeleton consistency, with the masked joints explicitly included in the loss so that missing-joint recovery and observed-joint stability were evaluated under the same protocol.

For fall-risk prediction, four representative baseline models were used.

CNN denotes an author-implemented temporal convolutional baseline consisting of three 1D convolutional layers (kernel size = 3), ReLU activation, max pooling, and a fully connected classification head.

Transformer denotes a standard temporal transformer encoder constructed using multi-head self-attention and feed-forward layers.

PoseFormer follows the original PoseFormer architecture proposed for skeleton-based spatial-temporal modeling.

DeGCN follows the decoupled graph convolutional network architecture for skeleton sequence analysis.

All fall-risk prediction baselines were trained and evaluated using the same training/validation/testing split, frame-level labels, and decision threshold selection protocol. The handcrafted stability features SFVR, TLA, and SV were not provided to these generic baselines; they were used only within StaFallNet. Therefore, the comparison with StaFallNet reflects the combined effect of stability-feature construction, feature fusion, and dynamic graph modeling.

### Details of implementation

The main implementation details and hyperparameter settings are summarized in Table 4.

**TABLE 4:** Main implementation details and hyperparameter settings.

Parameter	Value
Sequence length T	48 frames
GRU hidden dimension	256
Batch size (RePoseNet)	64
Batch size (StaFallNet)	32
Optimizer	Adam
Initial learning rate (RePoseNet)	0.001
Initial learning rate (StaFallNet)	0.0001
Temporal smoothness weight $\lambda_{\text{smooth}}$	0.1
Limb consistency weight $\lambda_{1}$	1.0
Symmetry constraint weight $\lambda_{2}$	0.5
Angle constraint weight $\lambda_{3}$	0.3
Task-aware weighting loss $\lambda_{4}$	1.0
Graph convolution layers	2
Graph hidden dimension	128
STFT window size	16 frames
Stride interval δ	10 frames
PCK thresholds	Defined in normalized pose coordinate space
Risk classification threshold	0.5
Temporal smoothing window	15 frames
Gaussian kernel parameter σ	0.8

**FIGURE 3:**
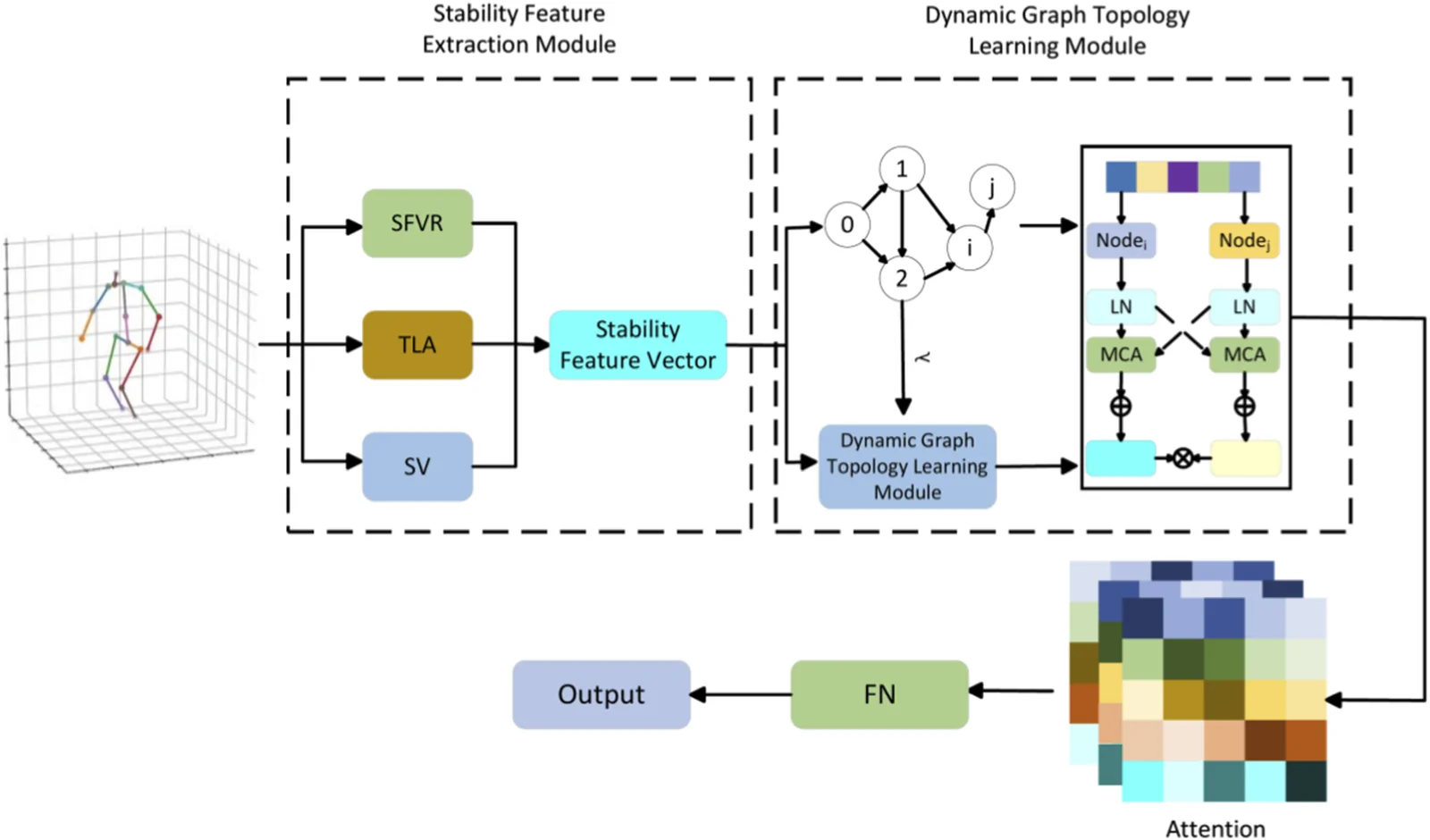
Overview of the StaFallNet architecture for real-time fall-risk prediction. The model consists of three modules: a stability feature extraction module, a dynamic graph topology learning module, and a fall-risk classification head. From the input 3D skeleton sequence, three biomechanical features—step frequency variation rate (SFVR), trunk leaning angle (TLA), and stride variability (SV)—are computed and fused into a stability feature vector. This guides the construction of a dynamic joint graph, which is refined through an attention-based mechanism before generating frame-level risk scores via the prediction network (FN).

All experiments were conducted using the PyTorch deep learning framework under Python 3.12.4 and CUDA 11.8 environments. For 3D pose estimation, we employed the proposed RePoseNet framework, which comprises a GRU-based temporal repair module followed by a biomechanics-constrained refinement stage. The input to RePoseNet consists of pseudo-3D skeleton sequences generated by the pretrained RTMPose-L + VideoPose3D pipeline. The resulting skeletons follow the COCO-17 joint format and are represented as frame-wise 3D joint coordinates. These 2D keypoints were normalized and fed into the temporal modeling module, where each GRU unit contains 256 hidden states, and the sequence length was fixed to T=48 frames. For the refinement stage, the joint coordinate consistency loss and bone-length regularization loss were jointly optimized. The overall architecture of StaFallNet is illustrated in Figure 3.

The model was trained on two NVIDIA RTX A100 GPUs with a batch size of 64 for 100 epochs. The Adam optimizer was adopted with an initial learning rate of 0.001 and a learning rate decay factor of 0.95 applied after each epoch. Data augmentation strategies included horizontal flipping and Gaussian noise perturbation on 2D keypoint inputs.

For the fall-risk prediction task, we constructed a stability-aware dynamic graph network based on the extracted 3D skeleton sequences. The input joint set followed the COCO-17 format, and stability indicators—including step frequency variation, trunk leaning angle, and stride variability—were concatenated as auxiliary features. The graph backbone was implemented using a dynamic topology GCN with two graph convolutional layers and temporal convolutions. Stability-aware adjacency matrices were dynamically updated based on joint-level relevance to fall-risk signals.

The classification head was composed of joint-wise attention pooling, followed by a fully connected layer with sigmoid activation for frame-wise risk prediction. Training was performed on a single NVIDIA RTX 4060 GPU using the Adam optimizer with an initial learning rate of 0.0001, which decayed by a factor of 0.1 at epochs 30 and 40. A total of 100 epochs were trained with a batch size of 32. All skeleton sequences were zero-centered and normalized before feeding into the model. Mini-batch stochastic gradient descent was used to ensure stable convergence.

To improve reproducibility, all experiments were conducted using a fixed random seed of 42. Model selection was performed according to the best validation F1-score. Early stopping was applied if the validation performance did not improve for 10 consecutive epochs.

## Results

### Reference-based skeleton refinement evaluation of RePoseNet

#### Benchmarking RePoseNet against baseline models

To evaluate the effectiveness of RePoseNet under a fair and unified skeleton-refinement setting, we benchmarked it against several representative temporal pose modeling and pose reconstruction baselines: Temporal CNN (TCNN), MHFormer (Li et al., 2022), STCFormer (Tang et al., 2023), and DTF (Ghafoor et al., 2024).

Although these methods were originally proposed for 3D pose estimation or spatiotemporal pose modeling, they were adapted in this study to the same pseudo-3D skeleton refinement task. Specifically, all models received identical corrupted pseudo-3D skeleton sequences and the corresponding binary visibility masks as input and were trained to reconstruct the corresponding pseudo-reference skeletons. The same train/validation/test split, artificial corruption protocol, optimization settings, and evaluation metrics were used for all methods. Consequently, the reported comparison evaluates reconstruction consistency under controlled skeleton corruption rather than end-to-end RGB-to-3D pose estimation performance. We report MPJPE and PCK with respect to pseudo-3D reference skeletons generated by the pretrained pose estimator. Since UCF101 does not contain true 3D joint annotations, these metrics are used to evaluate reference-based skeleton refinement consistency rather than absolute 3D reconstruction accuracy.

**FIGURE 4:**
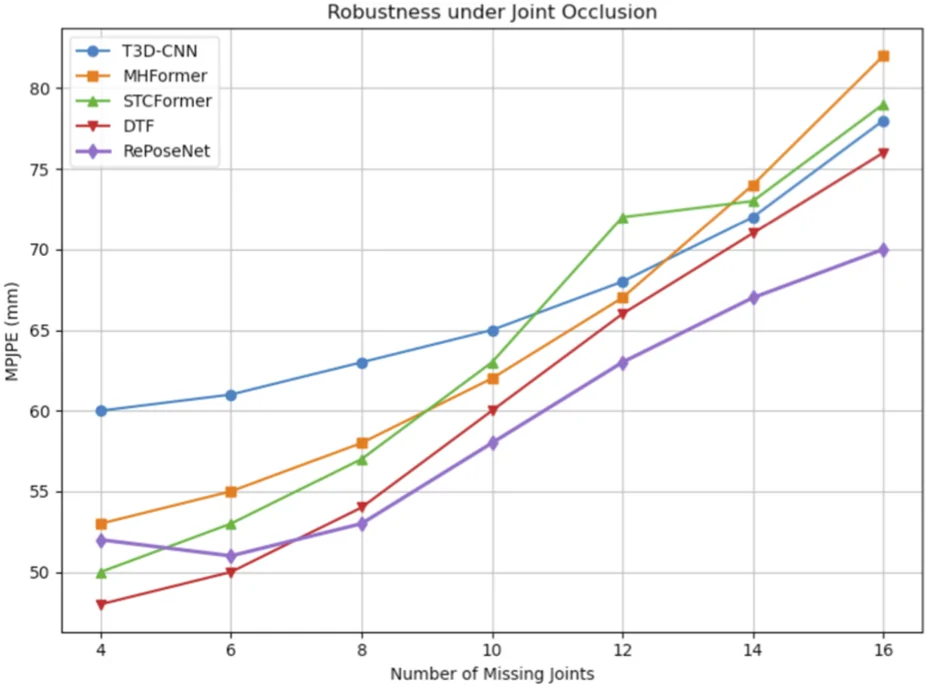
Robustness evaluation under artificial skeleton corruption. This figure illustrates the performance of various models, including Temporal CNN (TCNN), MHFormer, STCFormer, DTF, and RePoseNet, in terms of reference-based MPJPE (mm) computed against pseudo-3D reference skeletons. RePoseNet shows its clearest advantage under higher skeleton-corruption levels, while the low-corruption conditions show smaller differences among methods. The 16-missing-joint condition is interpreted as an extreme skeleton-level stress test that relies heavily on temporal interpolation and learned priors, not as proof of accurate pose recovery under near-complete image-level occlusion.

All baseline methods were adapted to the same pseudo-3D skeleton refinement setting and evaluated under identical artificial joint-corruption protocols. Therefore, the reported results reflect reconstruction consistency under controlled skeleton degradation rather than performance on the original pose-estimation tasks of the compared methods. To further investigate the robustness of RePoseNet under controlled skeleton corruption, we evaluate model performance under varying levels of artificial joint masking. Figure 4 presents the MPJPE results across five models—Temporal CNN (TCNN), MHFormer, STCFormer, DTF, and our proposed RePoseNet—as the number of missing joints increases from 4 to 16.

The curves show that RePoseNet’s advantage is most pronounced under higher skeleton-corruption levels. At lower corruption levels, especially the 4- and 6-missing-joint conditions, the margin over some adapted baselines is small and RePoseNet is not interpreted as uniformly superior at every point on the curve. As the number of missing joints increases, RePoseNet maintains lower reference-based MPJPE than the compared methods, indicating stronger reconstruction consistency with the pseudo-3D reference under severe controlled skeleton corruption.

It should be noted that these corruption levels were artificially generated at the skeleton level and do not necessarily reflect the frequency or structure of missing joints observed in real videos. In particular, the 16-joint corruption setting represents an extreme stress-test condition in which only one joint remains observed in the current frame. The result should therefore be interpreted as evidence that temporal interpolation and learned skeleton priors help recover skeletons close to the pseudo-reference under controlled corruption, rather than as evidence of accurate recovery under ordinary image-level occlusion or near-complete information loss.

Percentage of Correct Keypoints (PCK) is used here as a reference-based metric for evaluating skeleton refinement consistency. A refined joint is considered correct if its Euclidean distance from the corresponding pseudo-3D reference joint is within a specified threshold. In this context, PCK@20, PCK@30, and PCK@50 refer to thresholds of 20 mm, 30 mm, and 50 mm, respectively. Higher PCK values indicate stronger agreement with the reference skeletons and better robustness under joint corruption, but they should not be interpreted as accuracy against true motion-capture reference trajectory generated by the pretrained pose estimator.

**FIGURE 5:**
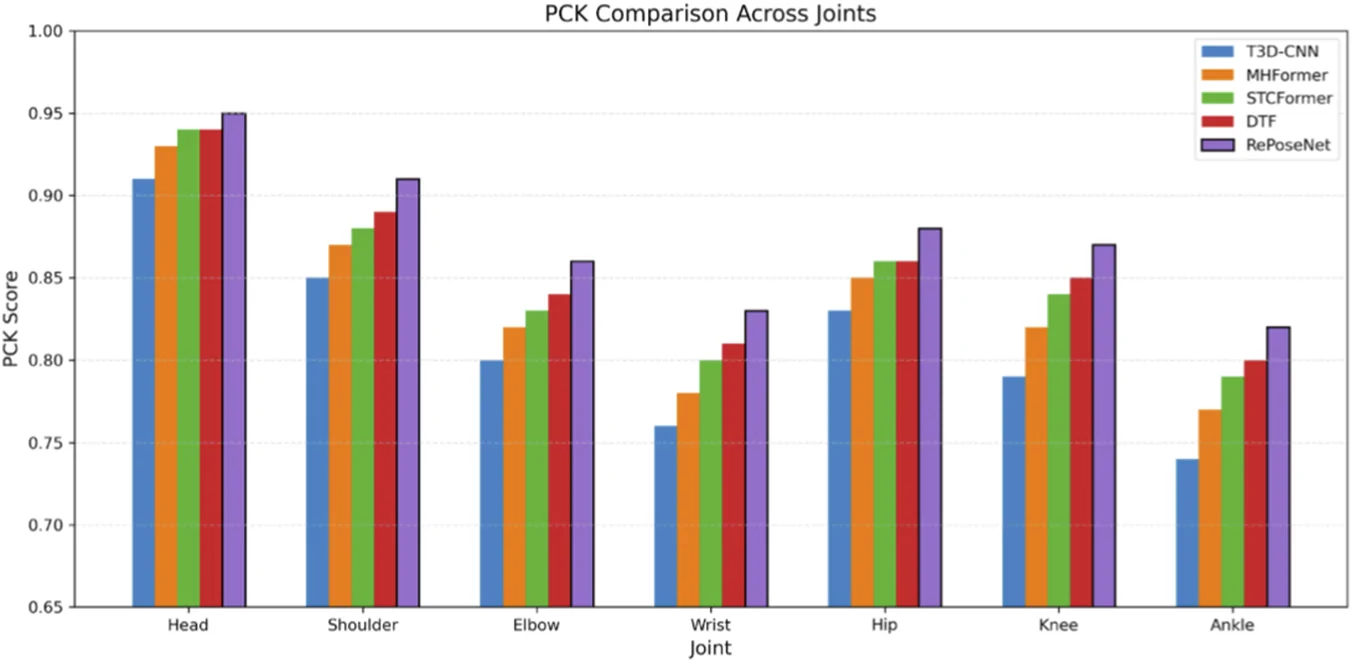
PCK comparison across joints. This figure presents the Percentage of Correct Keypoints (PCK) scores across seven major joints (Head, Shoulder, Elbow, Wrist, Hip, Knee, Ankle) for five models. RePoseNet achieves consistently higher PCK values across all joints, demonstrating superior reconstruction consistency and robustness, especially on distal joints such as Wrist and Ankle where accurate estimation is more challenging.

Table 5 and Figure 5 present a detailed comparison of PCK scores across seven anatomical joints. Our proposed RePoseNet consistently achieves the highest reference-based PCK across these reported joints, with an average PCK of 88%, notably outperforming baseline models such as Temporal CNN (TCNN) (81%), MHFormer (83%), STCFormer (85%), and DTF (86%).

**TABLE 5:** Reference-based PCK (%) comparison under different thresholds on the test set. PCK is computed with respect to pseudo-3D reference skeletons.

Method	PCK@20 ↑	PCK@30 ↑	PCK@50 ↑
Temporal CNN (TCNN)	62.8	74.5	88.2
MHFormer	67.1	78.3	91.0
STCFormer	69.4	80.1	92.7
DTF	70.9	82.2	93.8
RePoseNet (ours)	**74.5**	**85.7**	**95.2**

Bold values indicate the best performance.

Significant improvements are observed on distal joints like the Wrist and Ankle, which are typically more prone to occlusion and estimation errors. RePoseNet achieves a PCK of 0.83 on the Wrist and 0.82 on the Ankle, surpassing the second-best model (DTF) by 2–3 percentage points. These improvements highlight the model’s enhanced ability to capture fine-grained spatial dependencies and maintain temporal coherence.

In summary, RePoseNet demonstrates superior robustness in 3D skeleton refinement, especially under stricter evaluation criteria and challenging joint-corruption settings.

#### Temporal smoothness analysis of RePoseNet

To evaluate the temporal consistency of joint predictions, we further analyze the smoothness of the estimated trajectories across frames. Temporal smoothness is a critical factor in real-world applications such as gait monitoring and clinical motion analysis, where abrupt or jittery predictions may degrade downstream interpretability or diagnostic utility.

**FIGURE 6:**
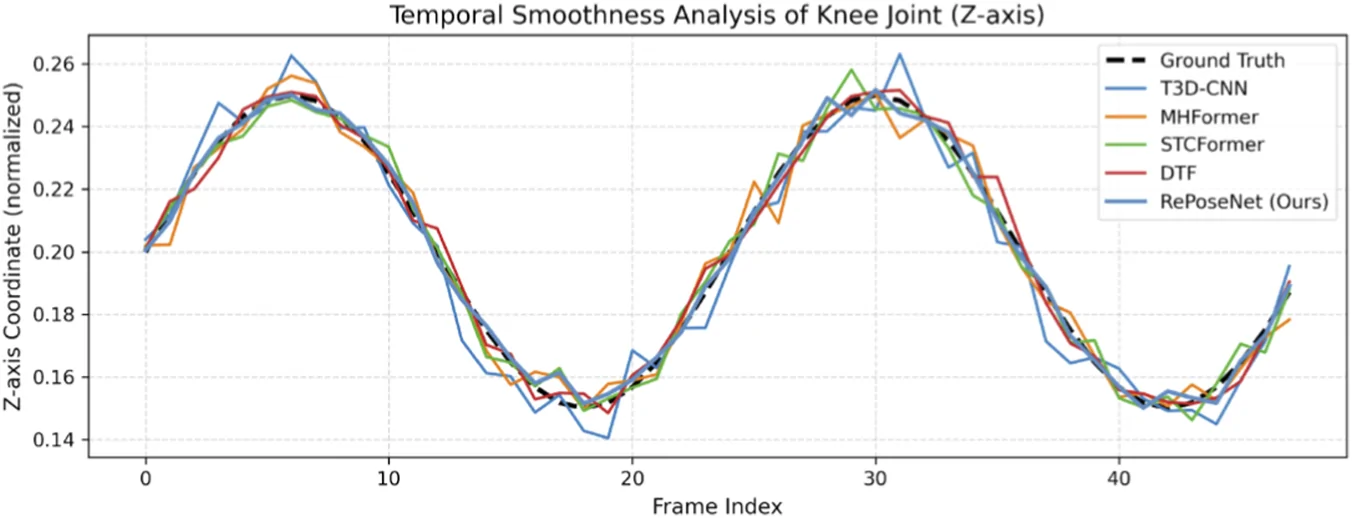
Temporal smoothness comparison of predicted knee joint trajectories along the Z-axis. Each line represents the Z-axis position over 50 consecutive frames for one subject. RePoseNet (blue) shows closer alignment with the reference trajectory generated by the pretrained pose estimator (dashed black) and exhibits smoother temporal dynamics compared to baseline models, indicating better temporal consistency and robustness.

Figure 6 illustrates the normalized Z-axis trajectories of the knee joint over 50 consecutive frames for one subject. The dashed black line represents the pseudo-3D reference trajectory generated by the pretrained pose estimator. , while the colored lines denote the outputs of different models. It can be observed that RePoseNet yields the most stable and smooth curve, closely following the reference trajectory generated by the pretrained pose estimator without introducing high-frequency fluctuations. In contrast, Temporal CNN (TCNN) and MHFormer exhibit evident jitter and deviation, particularly during phases of rapid motion change (e.g., around frames 10–15 and 30–35).

STCFormer and DTF produce moderately smooth curves but still suffer from transient discontinuities. In comparison, RePoseNet demonstrates superior temporal coherence, maintaining anatomical plausibility and eliminating oscillations across frames. This improvement stems from our cross-frame context modeling and trajectory-aware refinement module, which enables the model to learn stable motion patterns and suppress noise in occluded or ambiguous regions.

These results indicate that RePoseNet not only improves frame-level consistency but also ensures temporal smoothness of joint predictions, which is essential for high-fidelity biomechanical assessments and downstream temporal reasoning.

#### Robustness to noisy joint perturbations

To further distinguish missing-joint recovery from correction of erroneous but non-missing joint estimates, we conducted an additional robustness experiment using artificial coordinate perturbations.

Unlike the missing-joint corruption protocol, where selected joints are removed from the skeleton sequence, this experiment preserves all joint observations while introducing coordinate noise into the pseudo-3D skeleton trajectories. Specifically, Gaussian perturbations with different standard deviations were independently added to each joint coordinate:

$${\tilde{x}}_{t,j}=x_{t,j}+\epsilon_{t,j},\;\epsilon_{t,j}∼N\left(0,\sigma^{2}\right),$$

where $x_{t,j}$ denotes the original pseudo-3D joint coordinate and σ controls the noise magnitude.

Three perturbation levels ( σ=10 , 20 , and 30 mm) were evaluated. These perturbations simulate common pose-estimation errors such as coordinate bias, localization uncertainty, temporal jitter, and anatomically inconsistent joint displacements, while preserving the visibility of all joints.

The purpose of this experiment is fundamentally different from the missing-joint masking protocol. Whereas the masking experiment evaluates the ability of RePoseNet to reconstruct absent observations, the noisy-joint experiment evaluates its ability to suppress coordinate errors and restore anatomically plausible motion trajectories from corrupted but fully observed skeleton sequences.

Temporal smoothness was quantified using the normalized second-order temporal difference of the refined joint trajectory. For a refined skeleton sequence ${\hat{x}}_{t,j}$ , we first computed the acceleration-like temporal difference:

$$\Delta^{2}{\hat{x}}_{t,j}={\hat{x}}_{t+1,j}-2{\hat{x}}_{t,j}+{\hat{x}}_{t-1,j}.$$

The raw temporal variation was then calculated as:

$$E_{\text{temp}}=\frac{1}{\left(T-2\right)J}\sum_{t=2}^{T-1}\sum_{j=1}^{J}{∥\Delta^{2}{\hat{x}}_{t,j}∥}_{2}^{2}.$$

To make the values comparable across perturbation levels, we normalized this quantity by the mean temporal variation of the clean pseudo-reference skeletons, denoted as $E_{\text{ref}}$ , and reported:

$$\text{Temporal Smoothness}=\frac{1}{1+E_{\text{temp}}/\left(E_{\text{ref}}+\epsilon \right)}.$$

This score lies in $(\left.0,1\right]$ , where larger values indicate smoother trajectories and smaller high-frequency temporal fluctuations relative to the clean pseudo-reference skeletons.

Table 6 summarizes the performance of RePoseNet under increasing levels of coordinate perturbation.

**TABLE 6:** Robustness of RePoseNet under noisy joint perturbations.

Noise level	References MPJPE (mm) ↓	Temporal smoothness ↑	PCK@30 ↑
10 mm	49.1	0.903	0.952
20 mm	52.8	0.887	0.934
30 mm	57.4	0.861	0.911

Although performance gradually degrades as noise magnitude increases, RePoseNet maintains relatively stable reconstruction quality and temporal consistency across all perturbation levels. Even under the most severe condition ( σ=30 mm), the model preserves a high PCK value and produces smooth motion trajectories without introducing substantial temporal artifacts.

These findings suggest that RePoseNet is capable not only of recovering missing joints but also of correcting noisy and anatomically inconsistent joint estimates. This behavior is particularly important because real pose-estimation failures frequently manifest as coordinate inaccuracies, temporal jitter, left–right ambiguities, or implausible joint configurations rather than complete joint disappearance.

Therefore, the proposed framework provides robustness to both missing-joint corruption and noisy-joint perturbations, supporting its suitability as a skeleton refinement module for downstream stability-aware fall-risk prediction.

#### Ablation study on RePoseNet design components

To evaluate the individual contributions of each component in RePoseNet, we conducted a comprehensive ablation study by systematically removing key modules. This analysis aims to verify the effectiveness of the proposed architectural designs, including the Cross-Joint Attention Module, Residual Refinement Block, Temporal Alignment Module, and Gait-Aware Positional Encoding (GAPE).

Table 7 reports the performance variations across four evaluation metrics: MPJPE ↓ , PCK@20 ↑ , PCK@30 ↑ , and PCK@50 ↑ . The full model achieves the best reference-based refinement performance with the lowest MPJPE and the highest PCK scores, indicating improved consistency with the pseudo-3D reference skeletons under the same evaluation protocol. Notably, removing the Temporal Alignment Module leads to the most significant performance drop (MPJPE increases to 51.2 mm), highlighting its crucial role in capturing motion consistency across frames. Similarly, the exclusion of the Cross-Joint Attention Module or GAPE also results in noticeable degradation, especially in the PCK@20 and PCK@30 metrics, demonstrating the importance of spatiotemporal attention and pose-informed encoding.

**TABLE 7:** Ablation study on the contributions of different components in RePoseNet. The full model outperforms all variants, demonstrating the effectiveness of each module.

Model variant	MPJPE ↓	PCK@20 ↑	PCK@30 ↑	PCK@50 ↑
Full RePoseNet (ours)	**47.3**	**0.912**	**0.967**	**0.988**
w/o cross-joint attention	50.8	0.891	0.952	0.984
w/o biomechanical refinement	49.6	0.897	0.956	0.986
w/o GRU repair module	51.2	0.888	0.948	0.982
w/o gait-aware positional encoding	50.1	0.894	0.953	0.985

Bold values indicate the best performance.

**FIGURE 7:**
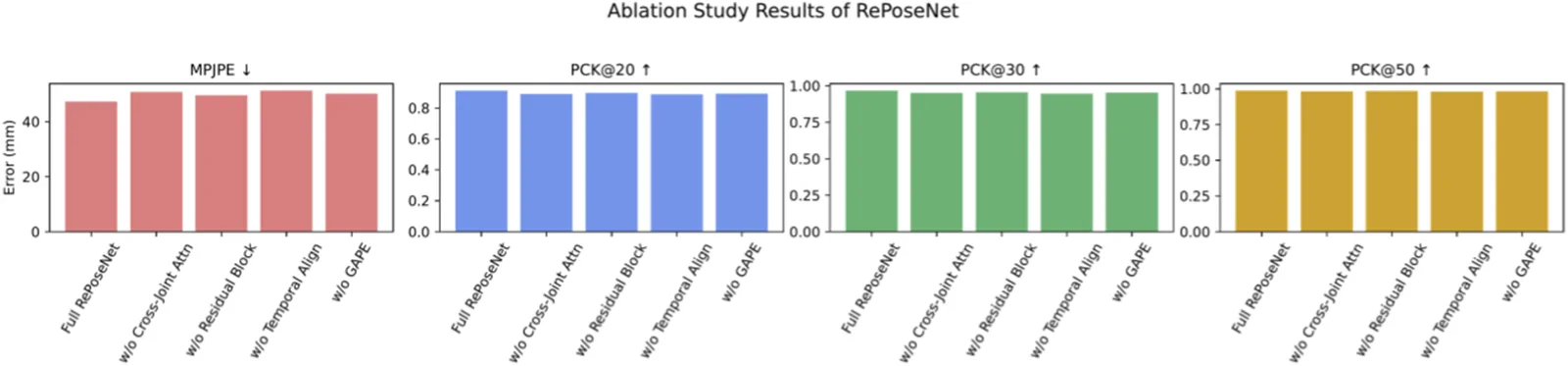
Ablation study results for RePoseNet. The figure presents a comparative analysis across four key evaluation metrics: MPJPE (Mean Per Joint Position Error), PCK@20, PCK@30, and PCK@50. Each subplot shows the effect of removing a specific module from the full model. The full RePoseNet achieves the best performance in all metrics, indicating the complementary contributions of the Cross-Joint Attention Module, Residual Refinement Block, Temporal Alignment Module, and Gait-Aware Positional Encoding (GAPE).

In addition to the numerical results, Figure 7 provides a visual comparison of the ablation variants across the four metrics. The MPJPE bar chart clearly shows that all variants underperform compared to the full RePoseNet, while the PCK metrics consistently follow a similar trend. These visualizations emphasize that each proposed module contributes positively to the model’s robustness and accuracy.

In summary, the ablation results confirm the necessity and effectiveness of each component in RePoseNet. Among all components, the Temporal Alignment Module contributes the largest performance gain, indicating that preserving temporal continuity is particularly important for instability-sensitive athletic motion analysis. The Cross-Joint Attention Module further improves spatial coordination modeling by capturing inter-joint dependencies, especially for distal joints that are easily corrupted under skeleton-level degradation. In addition, Gait-Aware Positional Encoding (GAPE) enhances movement-phase consistency and helps stabilize dynamic transitions. These results suggest that RePoseNet benefits primarily from the joint integration of temporal consistency modeling and biomechanically informed spatial coordination refinement, rather than from isolated architectural components alone. The full architecture benefits from the synergistic integration of spatial reasoning, temporal dynamics, and gait-aware positional priors, enabling more stable and biomechanically plausible 3D skeleton refinement from initially estimated pose sequences.

#### Effect of RePoseNet on downstream fall-risk prediction

To assess whether RePoseNet improves the input representation for StaFallNet, we compared four skeleton preprocessing strategies before the same downstream risk-prediction model. Raw Skeletons used the pseudo-3D skeletons generated by the RTMPose-L + VideoPose3D pipeline without additional preprocessing. Temporal Smoothing applied a 15-frame centered moving-average filter independently to each joint coordinate. Biomechanical Filtering applied deterministic anatomical correction rules, including limb-length stabilization using the sequence-level mean limb length, left–right symmetry regularization for paired limbs, and clipping of implausible joint-angle excursions to the physiological range used in the RePoseNet refinement loss. RePoseNet used the proposed learned temporal repair, cross-joint attention, and biomechanical refinement module. All variants were evaluated on the same held-out 43-event test set using the same StaFallNet architecture, threshold selection rule, and event-level early-warning protocol.

These results support the interpretation of RePoseNet as a skeleton refinement and preprocessing module rather than a standalone pose-estimation framework.

Compared with simple temporal smoothing and deterministic biomechanical filtering baselines, RePoseNet provides more reliable skeleton representations for downstream stability-aware risk prediction, with higher frame-level F1-score, higher event-level early-warning recall, and fewer false alarms.

The effect of different skeleton-preprocessing strategies on downstream fall-risk prediction is summarized in Table 8.

**TABLE 8:** Effect of different skeleton preprocessing strategies on downstream fall-risk prediction.

Method	Accuracy	F1-score	Event detection	False alarms
Raw skeletons	86.3	84.8	35/43 (81.4%)	0.18/min
Temporal smoothing	87.5	86.4	36/43 (83.7%)	0.16/min
Biomechanical filtering	88.1	87.1	37/43 (86.0%)	0.14/min
RePoseNet (ours)	**91.4**	**90.8**	**39/43 (90.7%)**	**0.11/min**

Bold values indicate the best performance.

### Performance of StaFallNet

#### Quantitative comparison with state-of-the-art baselines

To thoroughly evaluate the efficacy of StaFallNet, we benchmark it against four representative baseline models—CNN, Transformer, Poseformer, and DeGCN—covering both traditional and advanced architectures for skeleton-based sequence modeling. The evaluation was performed on the held-out event-level test set using the manually annotated frame-level pre-fall labels. Positive predictions therefore indicate detection of the pre-fall interval rather than recognition of frames after the fall event.

As shown in Table 9, StaFallNet achieves the best overall performance among the compared models, with an accuracy of 91.4% and an F1-score of 90.8%. Because SFVR, TLA, and SV were provided only to StaFallNet, this comparison should be interpreted as the performance of the complete stability-aware framework rather than as a pure architecture-only comparison. The observed gain stems from the combined effect of biomechanical feature extraction, feature fusion, and dynamic graph topology learning: the stability feature extraction module encodes transient movement abnormalities (e.g., abrupt rhythm variation, phase-inappropriate trunk tilt, and lower-limb coordination irregularity); the dynamic graph topology module allows frame-wise adaptive joint connectivity; and the classification head integrates stability-aware attention pooling and temporal decoding to forecast risk under noisy sports-video conditions.

**TABLE 9:** Performance comparison of StaFallNet and baseline models on fall-risk prediction. All metrics are reported as percentages except mAP. StaFallNet achieves the best performance across all metrics.

Model	Accuracy ↑	Precision ↑	Recall ↑	F1-score ↑	mAP ↑
StaFallNet (ours)	**91.4**	**88.6**	**93.1**	**90.8**	**0.912**
DeGCN	89.3	87.2	91.4	89.2	0.894
Poseformer	88.5	86.0	90.2	88.0	0.884
Transformer	87.2	84.5	89.1	86.7	0.867
CNN	85.3	82.4	87.7	84.9	0.849

Bold values indicate the best performance.

Because the number of non-risk frames was larger than the number of pre-fall frames, we used class-balanced sampling during training. In addition, the binary cross-entropy loss was weighted according to the positive-to-negative frame ratio to reduce bias toward the majority class.

In summary, StaFallNet’s overall performance supports the effectiveness of integrating domain-specific stability features with graph-based reasoning for predictive human motion analysis.

Further analysis indicates that stability-aware dynamic graph adaptation and handcrafted biomechanical stability indicators (SFVR, TLA, and SV) jointly contribute to StaFallNet’s performance. The graph module enables the model to focus on transient coordination disruptions between critical joints during unstable motion, while the handcrafted features provide complementary motion-stability cues under visually ambiguous or noisy pose sequences.

#### Event-level early warning analysis

Beyond frame-level classification accuracy, we further evaluated StaFallNet under an event-level early-warning protocol to assess its practical utility for pre-fall prediction.

Following the event-level train–test separation described in the dataset construction protocol, a fall event was considered successfully detected if the predicted risk score exceeded the decision threshold at least once within the predefined pre-fall interval before the fall onset. Event-level recall therefore measures the proportion of fall events for which the system generated an early warning prior to the actual fall.

Using a decision threshold of 0.5 and temporal smoothing over a 15-frame sliding window, StaFallNet successfully detected 39 of the 43 held-out test events before the visually identified fall onset, corresponding to an event-level detection rate of 90.7%. The average early-warning lead time was 0.82 s before fall onset. In addition, the false alarm rate was 0.11 false positives per video minute. The decision threshold of 0.5 was selected based on validation-set optimization using the best F1-score criterion. The event-level detection rate is therefore reported as the integer event-level proportion 39/43=90.7% .

These results indicate that the proposed framework is capable of detecting instability patterns before body-ground impact occurs, supporting its applicability to real-time sports safety monitoring and injury prevention scenarios.

#### Interpretable analysis of biomechanical relevance

To further bridge the proposed fall-risk prediction model with biomechanical theory and clinical application, we perform an interpretable analysis to identify key joints that contribute most significantly to risk discrimination. Specifically, we adopt a gradient-based attribution method to compute the saliency of each joint with respect to the model’s prediction confidence, thereby uncovering the underlying spatial attention pattern. For visualization purposes, the reported chest, spine, and pelvis regions correspond to virtual joints derived from the original COCO-17 skeleton according to the definitions provided in the Overall Pipeline section.

**FIGURE 8:**
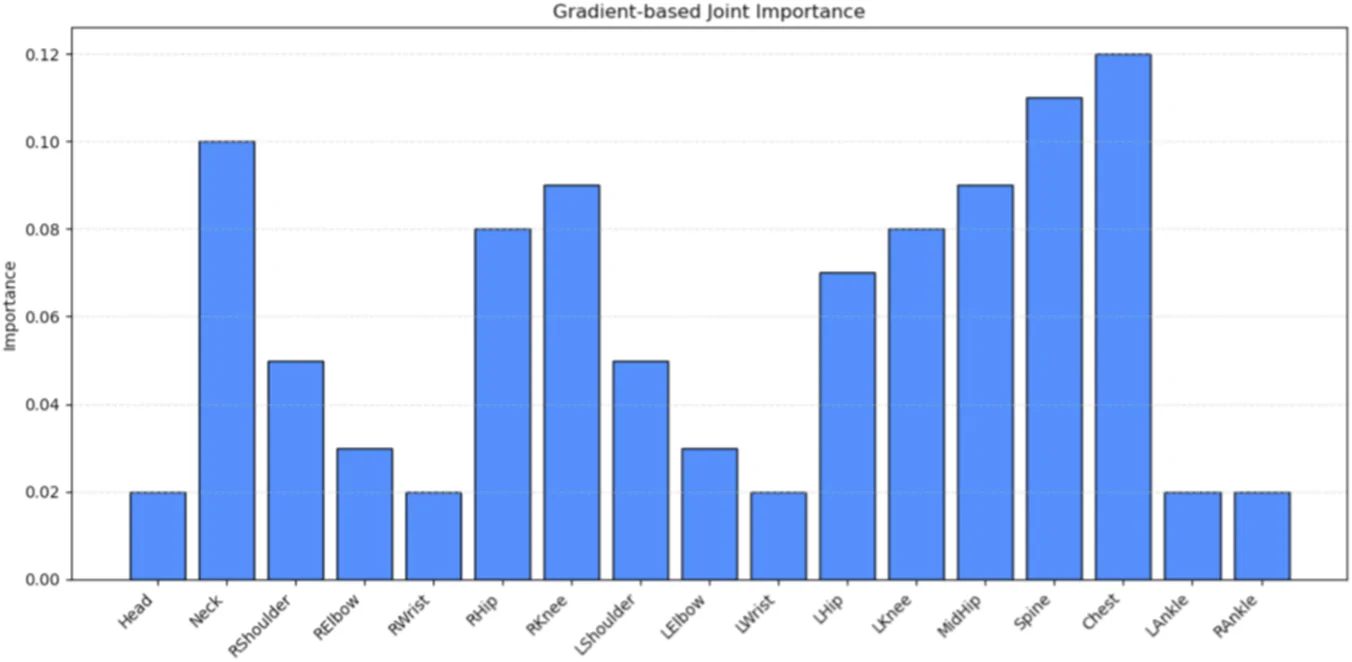
Gradient-based interpretability analysis of joint importance. The model assigns higher importance scores to the chest, spine, and pelvis regions, followed by the knees and hips, indicating their crucial roles in fall-risk discrimination. This result aligns with clinical understanding that trunk stability and lower-limb motion control are key indicators of pre-fall conditions.

As visualized in Figure 8, joints located in the central axis—such as the chest, spine, and pelvis—exhibit the highest importance scores, followed closely by lower-limb joints including the hips and knees. This spatial distribution suggests that the model emphasizes postural control and locomotor stability as essential cues for detecting imminent falls. Notably, distal upper-limb joints such as the wrists and elbows are assigned low relevance, supporting the notion that pre-fall instability predominantly originates from deficits in core-lower limb coordination.

These findings resonate strongly with established principles in sports science and kinesiology, particularly the “core–lower limb stability chain” theory. According to this view, effective force transmission and balance recovery rely on the integrated functioning of trunk stabilization and lower-limb control. The alignment between model attention and this theory indicates that StaFallNet not only achieves accurate predictions, but also reflects interpretable mechanisms grounded in physical performance theory. This enhances the model’s value in both clinical early warning systems and athletic training diagnostics.

## Discussion

This study proposes a two-stage deep learning framework consisting of RePoseNet for pseudo-3D skeleton refinement and StaFallNet for fall-risk prediction. RePoseNet is explicitly positioned as a post-estimation enhancement module that improves the temporal consistency and biomechanical plausibility of initially estimated skeleton sequences, while StaFallNet performs stability-aware dynamic graph modeling for frame-level risk assessment. Importantly, the primary contribution of this work is not the invention of entirely new neural network primitives, since GRUs, attention mechanisms, and graph convolutions are already established techniques. Instead, the novelty lies in the task-oriented reformulation and biomechanical integration of these components for athletic-motion pre-fall analysis. From the ablation results and comparative experiments, several key observations can be summarized. First, RePoseNet contributes mainly through temporal consistency enhancement and biomechanical plausibility refinement under controlled skeleton-level corruption, with the Temporal Alignment Module providing the largest individual improvement. Second, StaFallNet benefits from stability-aware dynamic graph adaptation, which enables the model to capture evolving inter-joint coordination breakdowns preceding falls. Finally, the incorporation of explicit biomechanical stability indicators (SFVR, TLA, and SV) improves interpretability and robustness by providing motion-stability cues beyond raw joint coordinates alone. Because these handcrafted indicators are used only within StaFallNet, the comparison reflects the complete stability-aware feature-fusion framework rather than the isolated effect of graph architecture alone. From a methodological perspective, the proposed framework can also be interpreted as a structured feature-fusion strategy for instability analysis. Instead of treating kinematic trajectories, stability descriptors, and graph representations independently, StaFallNet integrates these heterogeneous features into a unified spatial-temporal reasoning framework. This multi-level fusion design enables complementary interactions between explicit biomechanical priors and learned motion representations, thereby improving both predictive robustness and interpretability under complex athletic-motion conditions.

Existing methods typically use temporal modeling, graph reasoning, or pose smoothing independently for generic action recognition or pose estimation tasks. It should also be noted that the compared baseline methods were adapted to the controlled skeleton-refinement setting adopted in this work. Consequently, the reported comparisons evaluate their relative ability to recover corrupted pseudo-3D skeleton sequences rather than their original performance on full pose-estimation benchmarks. In contrast, the proposed framework explicitly couples temporal repair, biomechanical plausibility enforcement, and stability-aware graph adaptation within a unified instability-analysis pipeline. This enables the model to capture coordination breakdown patterns and anatomically inconsistent motion behaviors that are highly relevant to pre-fall states but are insufficiently addressed in existing skeleton-based frameworks.

The primary value of RePoseNet should be interpreted as improving the quality of skeleton representations used for downstream instability analysis rather than improving absolute pose-estimation accuracy. This interpretation is supported by the downstream ablation experiments, where RePoseNet consistently improves StaFallNet performance compared with raw skeleton inputs and simpler preprocessing alternatives.

We further acknowledge that UCF101 does not provide motion-capture-quality 3D annotations. Therefore, the pose-related evaluation in this study is based on pseudo-3D reference skeletons generated by the pretrained pose estimator. This means that MPJPE and PCK reflect consistency with the reference skeletons and the ability to maintain stable, anatomically plausible trajectories under corruption, rather than absolute 3D pose accuracy. We have moderated the corresponding claims throughout the revised manuscript to avoid overstating RePoseNet as a full 3D pose estimation model.

A further limitation concerns the choice of dataset. Although UCF101 provides challenging sports-motion videos, it was originally designed for action recognition rather than clinical fall-risk prediction. Therefore, the reported results should be interpreted as evidence of algorithmic feasibility for detecting visually annotated pre-fall patterns, rather than as medical-grade fall-risk assessment.

In addition, the generalization ability of the proposed framework to real-world sports or rehabilitation settings remains to be further validated. Future work should evaluate the framework on external datasets with clinically meaningful annotations, biomechanical measurements, and subject-independent testing protocols.

The complete StaFallNet framework achieves the highest accuracy (91.4%) and F1-score (90.8%) compared with representative baseline models including Transformer (Vaswani et al., 2017), PoseFormer (Zheng et al., 2021), and DeGCN (Myung et al., 2024). This improvement should be interpreted as the contribution of the integrated stability-feature extraction, feature fusion, and dynamic graph modeling strategy. Beyond performance metrics, gradient-based interpretability analysis reveals that the model assigns higher importance to the chest, spine, pelvis, and lower-limb joints (e.g., hips and knees), highlighting their dominant roles in motion stability and fall-risk discrimination.

This observation aligns with long-standing principles in sports science that emphasize the role of trunk–pelvis–lower-limb coordination in maintaining dynamic balance and reducing injury risk. For example, in sports actions such as squatting, jumping, and turning, core instability often leads to compensatory lower-limb patterns, increasing shear forces on the knee and disrupting hip–knee coordination. The model’s ability to autonomously identify these biomechanical keypoints without explicit guidance indicates that it learns not only discriminative but physiologically meaningful patterns, strengthening its potential for real-world deployment.

From a theoretical standpoint, the integration of RePoseNet and StaFallNet establishes a novel closed-loop pipeline linking motion quality to fall-risk prediction. RePoseNet provides stable and biomechanically plausible motion reconstruction, while StaFallNet performs fine-grained risk assessment using temporal pose dynamics. This framework is broadly applicable beyond fall detection—including athletic performance evaluation, motion quality scoring, and feedback-driven training interventions.

From a practical perspective, the proposed framework could support athlete monitoring in sports training and rehabilitation settings using ordinary RGB cameras. Coaches or clinicians may use the generated risk scores and joint-level attention patterns to identify unstable landing, trunk imbalance, or lower-limb coordination deficits during movement assessment.

Particularly in core stability assessment, functional strength evaluation, and movement screening, the ability of StaFallNet to highlight unstable movement tendencies supports the development of intelligent, theory-driven training prescriptions.

The reliability of StaFallNet depends on the operational definition of pre-fall risk. In this study, pre-fall frames were defined using a fixed temporal window before the visually identified fall onset. This definition provides a reproducible supervised learning target, but it may not capture all biomechanical pathways leading to falls. Future work should validate alternative labeling strategies, such as variable anticipation windows, event-level onset prediction, and expert-rated instability scores.

In addition to frame-level evaluation, we further assessed StaFallNet using an event-level early-warning protocol to better reflect practical deployment conditions. The model detected 39 of 43 held-out test events, generated risk alerts approximately 0.82 s before fall onset on average, and maintained a relatively low false alarm rate. Nevertheless, the current evaluation still relies on manually defined pre-fall windows and offline annotations. Future work should investigate continuous online prediction settings, adaptive anticipation horizons, and subject-independent real-world validation protocols.

Despite the promising results, limitations remain. The current models have not yet been validated in large-scale real-world sports environments, and the coupling between pose estimation and stability recognition warrants further optimization. Future work will focus on lightweight deployment, embedded system adaptation, cross-modal fusion (e.g., IMU, EMG), and domain generalization to enhance practical applications in sports training and educational settings.

Although the current framework demonstrates promising early-warning capability, computational cost and inference latency remain important challenges for large-scale real-time deployment. Future work will therefore investigate lightweight optimization and efficient streaming inference strategies for high-frame-rate sports environments.

Another important limitation concerns the scope of the defined fall-risk scenarios. The current framework is primarily designed for intrinsic instability patterns that emerge from impaired postural regulation, unstable landing mechanics, abnormal trunk control, or disrupted lower-limb coordination during athletic movement. Falls caused predominantly by unpredictable external perturbations—such as direct physical collisions, opponent interference, slippery surfaces, or sudden environmental disturbances—are not explicitly modeled in the current system because these events may not be inferable from body kinematics alone. Future work should incorporate interaction-aware modeling, environmental context analysis, and multimodal sensing to better address externally induced sports falls.

In addition, extreme camera viewpoints, severe occlusion, and rapid camera motion may reduce pose estimation quality and subsequently affect stability prediction performance. Future work will explore viewpoint-robust and multi-view modeling strategies for unconstrained sports scenarios.

## Conclusion

This work introduces a two stage framework that links anatomically consistent pose enhancement with stability aware prediction for early fall risk in athletic motion. The first stage refines initially estimated pseudo-3D skeletons generated by a pretrained RTMPose-L + VideoPose3D monocular pose estimation pipeline, producing temporally coherent and biomechanically plausible skeleton sequences under detector noise and controlled skeleton-level corruption. The second stage builds a dynamic joint graph, fuses stability indicators such as step frequency variation rate, Trunk Leaning Angle, and stride variability, and produces frame-level risk estimates. The pose-refinement results are evaluated against pseudo-3D reference skeletons rather than true motion-capture reference trajectory generated by the pretrained pose estimator, and therefore demonstrate improved skeleton consistency and robustness rather than absolute 3D pose reconstruction accuracy.

Rather than proposing isolated architectural novelties, the proposed framework contributes a biomechanically guided reformulation of skeleton refinement and dynamic graph reasoning for instability-aware athletic-motion analysis. This task-oriented integration distinguishes the framework from conventional pose enhancement or skeleton-based action recognition pipelines.

Together these results demonstrate a practical and interpretable path toward early warning of intrinsic movement-instability-related fall risk in competitive sports settings. The framework can support performance evaluation, targeted training, and injury prevention. Nevertheless, the current findings should be viewed as preliminary evidence obtained from a manually annotated subset of an action-recognition dataset. The framework has not yet been externally validated on clinical fall-risk datasets, prospective injury-prevention cohorts, or controlled biomechanical measurements. Future work will therefore prioritize validation on real-world sports and rehabilitation datasets, cross-dataset generalization, multimodal fusion with IMU or EMG signals, and prospective evaluation in on-field monitoring systems that provide timely and actionable guidance for athletes and coaches.

## Data Availability

Publicly available datasets were analyzed in this study. This data can be found here: https://www.crcv.ucf.edu/research/data-sets/ucf101/.

## References

[B1] Chen T. Xu D. Wang M. Zhou Z. Jie T. Zhou H. (2025). Wearable monitoring for rehabilitation: deep learning-driven vertical ground reaction force estimation for anterior cruciate ligament reconstruction. Clin. Biomech. 130, 106663. 10.1016/j.clinbiomech.2025.106663 40945379

[B2] Cho Y. Solak G. Nocentini O. Lorenzini M. Fortuna A. Ajoudani A. (2025). Anticipatory fall detection in humans with hybrid directed graph neural networks and long short-term memory. 10.48550/arXiv.2509.05337

[B3] Dindorf C. Horst F. Slijepčević D. Dumphart B. Dully J. Zeppelzauer M. (2024). “Machine learning in biomechanics: key applications and limitations in walking, running and sports movements,” in Artificial Intelligence, Optimization, and Data Sciences in Sports (Springer), 91–148.

[B4] Fukushima T. Blauberger P. Russomanno T. G. Lames M. (2024). The potential of human pose estimation for motion capture in sports: a validation study. Sports Eng. 27 (1), 19. 10.1007/s12283-024-00460-w

[B5] Ghafoor M. Mahmood A. Bilal M. (2024). Enhancing 3D human pose estimation amidst severe occlusion with dual transformer fusion. IEEE Trans. Multimedia 27, 1617–1624. 10.1109/tmm.2024.3521755

[B6] Han R. Yi M. Feng W. Qi F. Zhou Y. (2025). Enhancing accuracy in dynamic pose estimation for sports competitions using HRPose: a hybrid approach integrating SinglePose AI. Alexandria Eng. J. 127, 200–213. 10.1016/j.aej.2025.04.062

[B7] Hong S. Park S. (2025). Biomechanical optimization and reinforcement learning provide insight into transition from ankle to hip strategy in human postural control. Sci. Rep. 15 (1), 13640. 10.1038/s41598-025-97637-5 40254606 PMC12009993

[B8] Khoiee H. F. Z. Labbe D. Romeas T. Faubert J. Andrews S. (2025). “Multi-person physics-based pose estimation for combat sports,” in Proceedings of the Computer Vision and Pattern Recognition Conference, 5832–5841.

[B9] Leckey C. Van Dyk N. Doherty C. Lawlor A. Delahunt E. (2025). Machine learning approaches to injury risk prediction in sport: a scoping review with evidence synthesis. Br. J. Sports Med. 59 (7), 491–500. 10.1136/bjsports-2024-108576 39613453 PMC12013557

[B10] Lee C. Ahn J. Lee B.-C. (2025). The effects of perturbation intensities on backward slip-falls induced by a split-belt treadmill. Sci. Rep. 15 (1), 5108. 10.1038/s41598-025-89531-x 39934360 PMC11814418

[B11] Li W. Liu H. Tang H. Wang P. Van Gool L. (2022). “Mhformer: multi-hypothesis transformer for 3d human pose estimation,” in Proceedings of the IEEE/CVF Conference on Computer Vision and Pattern Recognition, 13147–13156.

[B12] Liu Li Xie H. Li X. Liao G. Wang S. Liao C. (2025). A real-time system for fall prediction and protection with spatio-temporal graph neural network using multiple motion sensors. Expert Syst. Appl. 281, 127549. 10.1016/j.eswa.2025.127549

[B34] Myung W. Su N. Xue J.-H. Wang G. (2024). DeGCN: deformable graph convolutional networks for skeleton-based action recognition. IEEE Trans. Image Process. 33, 2477–2490. 10.1109/TIP.2024.3378886 38526905

[B13] Robles Cruz D. Quiñones S. P. Belmar A. L. Figueroa D. Q. Hidalgo M. Toro C. T. (2024). Fall risk classification using trunk movement patterns from inertial measurement units and Mini-BESTest in community-dwelling older adults: a deep learning approach. Appl. Sci. 14 (20), 9170. 10.3390/app14209170

[B14] Sadr M. M. Khani M. Tootkaleh S. M. (2025). Predicting athletic injuries with deep learning: evaluating CNNs and RNNs for enhanced performance and safety. Biomed. Signal Process. Control 105, 107692. 10.1016/j.bspc.2025.107692

[B15] Schoeneberg B. McNeal B. Reisner J. Friesen A. Reed T. Goodwin J. (2024). The efficacy of perturbation-based balance training among older adults: a systematic review. Phys. and Occup. Ther. Geriatrics 42 (4), 339–355. 10.1080/02703181.2024.2319241

[B16] Shin S. Simpkins C. Ahn J. Yang F. (2024). Impact of standing perturbation intensities on fall and stability outcomes in healthy young adults. J. Biomechanics 168, 112123. 10.1016/j.jbiomech.2024.112123 38696984

[B17] Shokouhi S. Sritharan P. Lee P.V.-S. (2024). Recovering whole-body angular momentum and margin of stability after treadmill-induced perturbations during sloped walking in healthy young adults. Sci. Rep. 14 (1), 4421. 10.1038/s41598-024-54890-4 38388724 PMC10884438

[B18] Soomro K. Zamir A. R. Shah M. (2012). Ucf101: a dataset of 101 human actions classes from videos in the wild. arXiv Prepr. arXiv:1212.0402. 10.48550/arXiv.1212.0402

[B19] Sugiarto T. Lin Y.-J. Tsai H.-L. Sun C.-T. Hsu W.-C. (2025). Performance of deep-learning models incorporating knee alignment information for predicting ground reaction force during walking. Biomed. Eng. OnLine 24 (1), 78. 10.1186/s12938-025-01409-1 40551106 PMC12186330

[B20] Tanaka R. Suzuki T. Fujii K. (2024). “3d pose-based temporal action segmentation for figure skating: a fine-grained and jump procedure-aware annotation approach,” in Proceedings of the 7th ACM International Workshop on Multimedia Content Analysis in Sports, 17–26.

[B21] Tang Z. Qiu Z. Yanbin H. Hong R. Yao T. (2023). “3d human pose estimation with spatio-temporal criss-cross attention,” in Proceedings of the IEEE/CVF Conference on Computer Vision and Pattern Recognition, 4790–4799.

[B22] Vaswani A. Shazeer N. Parmar N. Uszkoreit J. Jones L. Gomez A. N. (2017). Attention is all you need. Adv. Neural Inf. Process. Syst. 30, 5998–6008. 10.48550/arXiv.1706.03762

[B24] Wang S. Li X. Liao G. Liu J. Liao C. Liu M. (2024a). A spatio-temporal graph neural network for fall prediction with inertial sensors. Knowledge-Based Syst. 293, 111709. 10.1016/j.knosys.2024.111709

[B25] Wang Z. Xie H. Chien J. H. (2024b). The margin of stability is affected differently when walking under quasi-random treadmill perturbations with or without full visual support. PeerJ 12, e16919. 10.7717/peerj.16919 38390385 PMC10883149

[B26] Woomin M. (2023). “DeGCN: decoupled graph convolutional network for skeleton-based action recognition,” in Proceedings of the AAAI Conference on Artificial Intelligence.

[B27] Xi X. Zhang C. Jia W. Jiang R. (2024). Enhancing human pose estimation in sports training: integrating spatiotemporal transformer for improved accuracy and real-time performance. Alexandria Eng. J. 109, 144–156. 10.1016/j.aej.2024.08.072

[B28] Xu D. Zhou H. Jie T. Zhou Z. Yuan Yi Jemni M. (2025). Data-driven deep learning for predicting ligament fatigue failure risk mechanisms. Int. J. Mech. Sci. 301, 110519. 10.1016/j.ijmecsci.2025.110519

[B29] Xu D. Zhou H. Yuan Yi Zhang Z. Jie T. Zhou Z. (2026). Data-driven computing ligament loading mechanisms: integration of the computational ligament mechanics models with deep learning. Acta Mech. Sin. 42 (5), 625304. 10.1007/s10409-025-25304-x

[B30] Yeung C. Suzuki T. Tanaka R. Yin Z. Fujii K. (2025). “AthletePose3D: a benchmark dataset for 3D human pose estimation and kinematic validation in athletic movements,” in Proceedings of the Computer Vision and Pattern Recognition Conference, 5945–5956.

[B31] Yin Z. Yeung C. Suzuki T. Tanaka R. Fujii K. (2025). KASportsFormer: kinematic anatomy enhanced transformer for 3D human pose estimation on short sports scene video. 10.48550/arXiv.2507.20763

[B32] Zheng S. Zhang T. Wang W. Li L. Yu N. Qiao Yu (2021). “PoseFormer: 3D human pose estimation with spatial-temporal transformers,” in Proceedings of the IEEE International Conference on Computer Vision.

[B33] Zhou Z. Xu D. Jie T. Wang M. Zhang Z. Zhou H. (2026). Enhanced identification of chronic ankle instability under different conditions: a new evaluation framework based on feature fusion and machine learning. IEEE Trans. Neural Syst. Rehabilitation Eng. 34, 1318–1328. 10.1109/TNSRE.2026.3667526 41734114

